# In Silico Verification of Predicted Potential Promoter Sequences in the Rice (*Oryza sativa*) Genome

**DOI:** 10.3390/plants12203573

**Published:** 2023-10-14

**Authors:** Anastasiya N. Bubnova, Irina V. Yakovleva, Eugene V. Korotkov, Anastasiya M. Kamionskaya

**Affiliations:** Federal State Institution Federal Research Centre «Fundamentals of Biotechnology», Russian Academy of Sciences, 119071 Moscow, Russiaakamio@fbras.ru (A.M.K.)

**Keywords:** promoter, rice, *Oryza sativa*, predicted sequences, transcription, regulatory elements of genome

## Abstract

The exact identification of promoter sequences remains a serious problem in computational biology, as the promoter prediction algorithms under development continue to produce false-positive results. Therefore, to fully assess the validity of predicted sequences, it is necessary to perform a comprehensive test of their properties, such as the presence of downstream transcribed DNA regions behind them, or chromatin accessibility for transcription factor binding. In this paper, we examined the promoter sequences of chromosome 1 of the rice *Oryza sativa* genome from the Database of Potential Promoter Sequences predicted using a mathematical algorithm based on the derivation and calculation of statistically significant promoter classes. In this paper TATA motifs and cis-regulatory elements were identified in the predicted promoter sequences. We also verified the presence of potential transcription start sites near the predicted promoters by analyzing CAGE-seq data. We searched for unannotated transcripts behind the predicted sequences by de novo assembling transcripts from RNA-seq data. We also examined chromatin accessibility in the region of the predicted promoters by analyzing ATAC-seq data. As a result of this work, we identified the predicted sequences that are most likely to be promoters for further experimental validation in an in vivo or in vitro system.

## 1. Introduction

Rice is a tropical plant from of the genus of annual and perennial herbaceous plants of the grass family; a cereal species. Lots of nations treat it as a second bread. According to the time of cultivation and its valuable qualities, it is rightfully considered the most popular cereal in the world. 

Due to its high agricultural importance, the problems and risks associated with rice cultivation are under the observation of scientists in a wide range of fields, from agronomic sciences to molecular biology and biotechnology. In the field of agronomy, different approaches to rice cultivation are being explored, and there is active research into the genetic differences between domesticated rice species and their wild relatives in order to develop loci that can be used to create a new generation of resistant rice varieties. Molecular biologists and biotechnologists are exploring the possibility of the genomic editing of rice to improve its nutritional properties and increase its environmental resilience [1].

The rice genome is quite well studied, sequenced, and annotated. In 2005, the International Rice Genome Sequencing Project (IRGSP) group provided, for the first time, a reference rice genome sequence covering 95% of the *O. sativa* Nipponbare genome [2]. Later, the RAP-DB Rice Genome Annotation Project database (available at https://rapdb.dna.affrc.go.jp/, accessed on 20 May 2022) [3] and a database based on the structural and functional annotation of the rice genome based on pseudomolecules were created [4]. In addition, other informative resources and tools for working on the rice genome were actively developed and appeared, with the exception of programs and algorithms for predicting regulatory elements of the rice genome, in particular promoter sequences, and databases for storing information about them.

A promoter is a sequence of DNA nucleotides in the vicinity of the transcription start site, necessary for the formation of the preinitiator complex. The length of promoters usually varies from 100 to 1000 bp; it can vary significantly from gene to gene. Eukaryotic promoters are usually divided into three regions: core/basic, distal, and proximal regions [5]. The core or core promoter is the smallest promoter region that is capable of initiating transcription and is required for assembly of the pre-initiation complex (PIC); it typically includes a transcription start site (TSS) and is located at a distance of −60 to +40 bp from the TSS [6]. Also, many core promoters contain the TATA box, which is located 25–45 bp upstream of the TSS and is a conserved DNA sequence (5′-TATAAA-3′) where common transcription factor proteins can bind. The TATA box is an important functional signal in eukaryotic promoters and, in some cases, can independently direct the precise initiation of transcription by RNA polymerase II even in the absence of other transcriptional elements [7]. Many promoters of highly expressed genes contain a TATA box in their core region, however, there are also large groups of highly expressed genes in which the TATA box is absent in the promoters, for example: housekeeping and photosynthesis genes. This means that the presence of the TATA box in the core promoter region is not a mandatory criterion for promoter function [8]. The proximal region of the promoter is located ~250–1000 bp upstream of the main promoter. The proximal promoter usually contains several transcription factors binding sites that are responsible for the specific regulation of transcription. The distal promoter regions can also contain transcription factor binding sites, but these regions mainly contain regulatory elements: enhancers, silencers, and insulators. The distal promoter, together with regulatory elements, is often required for the accurate reproduction of expression patterns, and distal cis-regulatory elements can be located in introns, which can make the computer study of these regions difficult.

Proper identification of promoter sequences plays an important role in understanding the dynamics, patterns, and regulation of gene expression. In addition, when genome editing or combining DNA fragments into new synthetic sequences, it is critical for predicting the potential formation of new promoter sequences. In recent years, a great abundance of bioinformatics tools have been created to predict promoter sequences in the genome or in a DNA region of interest. But the exact identification of promoter sequences remains a major challenge in computational biology, as even the best algorithms can generate false results that are indistinguishable from true ones.

To detect in the genome, and characterize and quantify the activity of promoters, various sequencing-based analysis methods have been developed, such as: CAGE (gene expression cap analysis), which allows for measuring the expression of eukaryotic capped RNAs and simultaneously displaying promoter regions [9]; PRSeq (promoter RNA sequencing), which is a massive and quantitative method for analyzing the specificity and strength of the promoter, based on the creation of a template DNA pool that carries information about its own promoter in its transcribed region [10]; PEAT (analysis of paired ends of transcription start sites), based on the mapping of transcription initiation patterns using paired-end sequencing [11]; and RAMPAGE (RNA annotation and mapping of promoters for analysis of gene expression), which is a method that uses highly specific sequencing of 5′-complete complementary DNA to identify transcription start sites (TSS) throughout a genome [12].

When predicting promoter sequences in silico using computational biology methods, developers mainly use the following strategies for predicting and identifying regulatory elements: the use of scoring functions, machine learning-based algorithms, and deep learning-based approaches.

In 2019, Meng Zhang and his team compared and evaluated computational promoter prediction tools developed from 2000 to 2019 [13]. After analyzing 58 datasets and 19 predictors, the scientists concluded that estimator-based approaches are the easiest to implement, but usually have a lower performance than other approaches. Deep learning approaches tend to be more time-consuming and computationally intensive, but achieve very good results in predictive performance. Traditional methods based on machine learning are more balanced in terms of computational load and algorithm complexity. The researchers also concluded from their analysis that scoring tools tend to predict more false positives or have low sensitivity when applied to predefined promoter sequences. However, the prediction results from these methods can still be useful for some meaningful biological inferences.

In this work, we studied the sequences of the first chromosome of the *O. sativa* rice genome from the Database of Potential Promoter Sequences (available online at http://victoria.biengi.ac.ru/cgi-bin/dbPPS/index.cgi, accessed on 20 August 2022), predicted to be promoter elements using a mathematical sequence prediction method based on multiple alignments [14]. Multiple alignments were created using the MAHDS method [15], a mathematical method for calculating multiple alignments for highly divergent sequences. When searching for promoters using computerized methods, the problem of the number of false-positive results arises. The number of false positives is usually high and often devalues all the results obtained via computer methods because it is difficult to understand where the noise is and where there is really a signal. When using the MADHS method the number of false positives for the randomly mixed rice genome is less than 10^−8^ per nucleotide [14]. Generally, the number of false positives in promoter sequence searchers is at least 10^−4^ per nucleotide. Therefore, all other theoretical databases are at least four orders of magnitude worse than this database, because the number of false positives is less than 10^−8^ for this database. Thus, the potential promoters included in the database we used are practically unaffected by random processes. In this sense, the results of this database are the most promising for experimental verification.

## 2. Results

Firstly, all selected promoters under study were analyzed for the absence of an intersection with annotated promoters and transcripts within 1000 bp downstream of the annotated transcripts.

Next, TATA-motifs regions were identified in the selected promoters under study (Table S1). Also, cis-regulatory elements were identified in the putative promoter region (Table S1). The identified cis-regulatory elements are involved in abscisic acid responsiveness; low-temperature responsiveness; MeJA-responsiveness; anaerobic induction; gibberellin-responsiveness; phytochrome down-regulation expression; light responsiveness; auxin responsiveness; and meristem expression.

### 2.1. Investigated Predicted Promoters vs. Predicted Promoters from Another Database

As a result of comparing the studied promoters from the Database of Potential Promoter Sequences with the promoters from the PlantRegMap database, it was found that only nine of the studied promoters—14, 15, 29, 51, 80, 90, 92, 105, and 116—intersect with the promoters from PlantRegMap. This may indicate the correctness of the prediction of the function of these sequences, since they were predicted to be promoters by two different independent algorithms.

### 2.2. Potentially New Transcription Start Sites behind the Investigated Predicted Promoters

**Figure 1:**
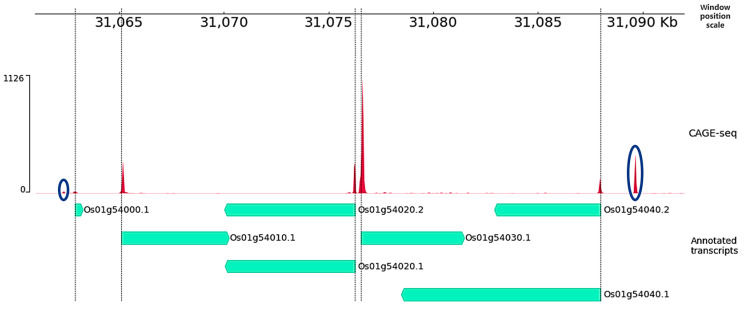
Rice genome fragment. Mint lines with direction are genes from the *O. sativa* MSU Release 7.0 rice genome annotation. Red peaks are TSS mapped to the genome from DRR127211 CAGE-sequencing data. Blue indicates peaks of potential unannotated TSS. Visualized with the help of PyGenomeTracks [16].

While analyzing the CAGE-seq data, we found CAP sites mapped to regions of the rice genome free of known annotated genes, as shown in Figure 1 and highlighted in blue. This may indicate the possible existence of previously unannotated transcripts in the rice genome. Also in this figure, it can be observed that the transcription start sites of the annotated genes (mint color—“Annotated transcripts”) occur with the peaks (red color) obtained by analyzing the CAGE-seq data. This confirms the validity of our analysis of the cap-expression data. And it suggests the presence of an unannotated transcription in those regions of the genome where there are noticeable peaks in the cap analysis and no annotated sequence data.

**Figure 2:**
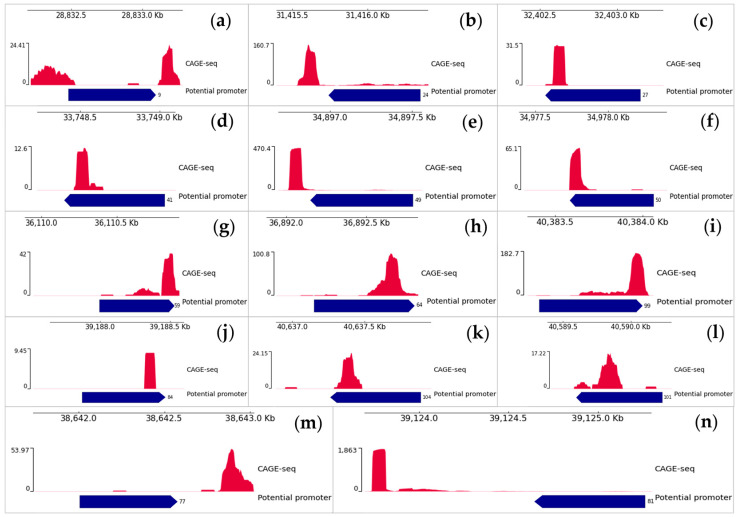
All blocks of the figure represent the result of a search for potential transcription start sites by analyzing CAGE-seq data. Each block represents information about one promoter (indicating the serial number of the promoter): (**a**)—9; (**b**)—24; (**c**)—27; (**d**)—41; (**e**)—49; (**f**)—50; (**g**)—59; (**h**)—64; (**i**)—99; (**j**)—84; (**k**)—104; (**l**)—101; (**m**)—77; (**n**)—81. Promoter sequences are presented in the form of dark blue lines with their Sr. No. and with a direction (which indicates the location of the promoter on the + or − strand of the DNA). Red peaks represent sequences of the 5′ ends of capped eukaryotic RNAs aligned to the rice genome (CAGE-sec data analysis), which represent potential TSS. At the top of each picture block the window position scale in *O. sativa* genome is indicated.

Among such unannotated potential transcription start sites there are also those located close to some of the investigated predicted promoters (Figure 2), among them:

- Three potential TSS located no further than 100 bp from the 3′-end of the predicted promoters (promoter numbers: 9, 24, 49);
- Nine potential TSS overlapping with the 3′-end of the promoter (promoter numbers: 27, 41, 50, 59, 59, 64, 84, 99, 101, 104);
- One potential TSS located no further than 300 bp from the 3′-end of the predicted promoter (promoter number: 77);
- One potential TSS that has a distinct peak located no further than 1000 bp from the 3′-end of the predicted promoter (promoter number 81) and lower peaks, the closest of which is no further than 250 bp from the 3′-end of the promoter.

Thus, these predicted promoters may indeed be the promoter region of the genome from which transcription starts, as there are potential unannotated transcription start sites behind them. 

### 2.3. De Novo Assembled Non-Annotated Transcripts

We then searched for unannotated transcripts in the rice genome via de novo genome assembly. First, we selected transcripts located up to 1000 bp in the 3′ (*downstream*) direction from predicted promoter sequences. However, few transcripts were found in such a range, so we decided to extend the search range to 5000 bp. Theoretically, promoters can be distanced from genes by embedding mobile genome elements. Therefore, we thought it would be interesting to study the assembled transcripts more distant from the potential promoters as well. 

As shown in Table 1, 10 out of the 1825 assembled transcripts are within 5000 bp of the studied promoters. The length of the selected de novo transcripts ranges from 220 to 860 bp. All these transcripts have open reading frames—ORFs (the fragments of DNA sequences between start and stop codons). Some transcripts have several ORFs; only the maximum frame lengths are indicated in the table. Quantitative analysis of gene expression based on the normalization of these transcriptome samples reveals the presence of an expression (TPM—the number of transcripts per million mapped reads), which varies in different transcripts from 10 to ~1000. 

The results of the homology search are presented in Table 2. In tomato, homologous proteins were not found. In *Arabidopsis*, two molecules of the photosystem were found behind the 37th promoter: 5MDX and, with a smaller degree of homology, 7OUI. Also with a small percentage of coverage and identity in *Arabidopsis*, the 6KKS protein was found, which belongs to the transcription factors MYB family and plays an important role during plant growth and development. Identity details the percentage of base pairs or amino acids that match between the sequence of the assembled transcript and the reference sample. Query cover indicates the percentage of the length of the query sequence included in the alignment. This protein is involved in two processes: primary carbon dioxide fixation and fragmentation of the pentose substrate during photorespiration.

### 2.4. Investigated Predicted Promoters vs. Predicted Promoters from Another Database

**Figure 3:**
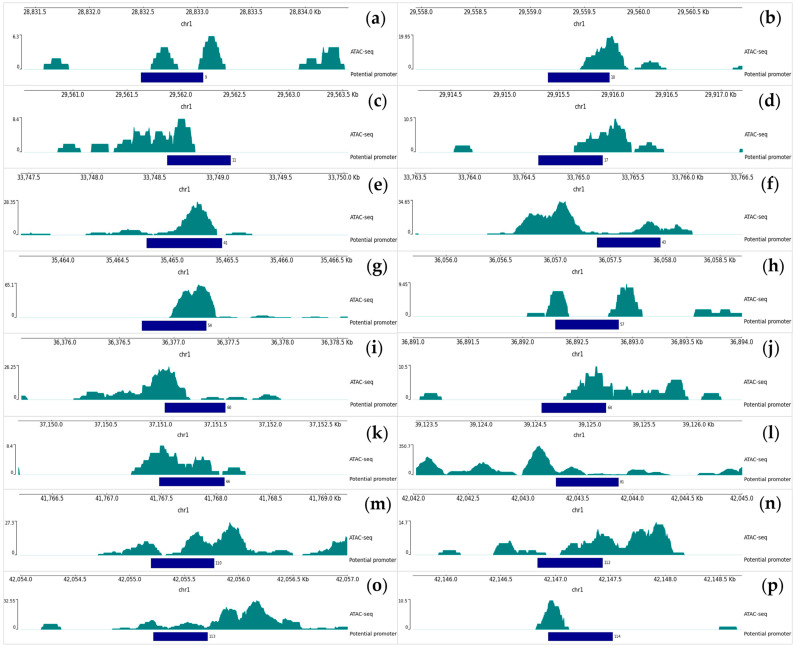
All blocks of the figure represent the result of chromatin accessibility analysis (ATAC-seq). Each block represents information about one promoter (indicating the serial number of the promoter): (**a**)—9; (**b**)—10; (**c**)—11; (**d**)—17; (**e**)—41; (**f**)—43; (**g**)—54; (**h**)—57; (**i**)—60; (**j**)—64; (**k**)—66; (**l**)—81; (**m**)—110; (**n**)—112; (**o**)—113; (**p**)—114. Promoter sequences are presented in the form of dark blue lines with their Sr. No. and with a direction (which indicates the location of the promoter on the + or − strand of the DNA). Teal peaks represent sequences located in an area of open chromatin. At the top of each picture block the window position scale in *O. sativa* genome is indicated.

We also analyzed chromatin accessibility in the region of the predicted promoter sequences by analyzing ATAC-seq data. As a result, we selected 16 predicted promoters (Figure 3) that overlap with ATAC-seq peaks. This indicates that the predicted promoters are located in open regions of chromatin that are accessible for transcription factor seeding. 

As a result, we can distinguish four predicted promoters, in which several factors characteristic of promoter sequences were identified simultaneously via in silico analysis. Promoters 9, 41, 64, and 81 are located in an open region of chromatin, which physically allows transcription factors to bind to them, and these predicted promoters also have transcription behind them that has not been annotated earlier. 

## 3. Discussion

To predict new promoter sequences in the rice genome, we used the previously described mathematical sequence prediction method based on multiple alignments (MAHDS). But at the moment, the occurrence of false-positive results in sequence prediction remains an unsolved problem in all computational biology, including the method we use, in which the number of false positives for a randomly mixed rice genome is <10^−8^ per nucleotide. In this regard, we consider it necessary to conduct additional studies of the predicted sequences to confirm their functional role in the genome. And in our opinion, the obligatory completion of the verification of the functioning of predicted promoters should be the experimental verification of them in vivo or in vitro.

We saw that prediction tools based on different algorithms—MAHDS and an algorithm based on searching for DNA-binding domains of transcription factors—produced predominantly divergent predictions of promoter regions of the rice genome. As we can see from the results, only 9 of the studied 126 promoters predicted via the MAHDS method were also detected in the Plant Reg Map database based on another prediction algorithm. Can we be sure that these nine sequences are really promoters since each of them was predicted via different independent methods? Probably, yes. However, as we can see from our results, we did not detect unannotated TSSs or transcripts behind any of these nine predicted promoters. We also did not see accessible open chromatin in the region of these sequences. 

It is well known that the accessible region of chromatin does not physically prevent the binding of transcription factors, which is important to consider when studying the properties of putative promoter sites. In our study, we paid attention to investigating the issue of the degree of chromatin openness in the neighborhood of predicted regulatory sequences. As a result, we obtained a number of predicted promoters located in the open region of chromatin (Figure 3)—16 sequences. At this time, we do not have data that can prove the location of the other predicted promoters in the region available for transcription factor seeding. However, this cannot exclude the other predicted sequences from the range of potential promoters. It would seem that the predicted sequences located in the region of closed chromatin cannot be functional promoters. But there is evidence that certain transcription factors can bind to closed chromatin and recruit chromatin remodeling factors to open chromatin, providing Pol II binding and the initiation of transcription [17,18]. To better know the nucleosome-free regions of the rice genome, the results of other rice genome sequencing methods can also be studied in the future. For example, FAIRE-Seq (Formaldehyde-Assisted Isolation of Regulatory Elements), DNase-Seq (sequencing regions sensitive to cleavage by DNase I), and MNase-seq (micrococcal nuclease digestion with deep sequencing). 

The presence of a promoter in the genome implies the presence of transcription behind it. One of the options for finding promoters or confirming the role of predicted promoters is to search for transcripts or transcription start sites. In this work we used both strategies. It is worth noting that the TSS search gave us more results, which showed the presence of 5′-end transcripts directly near the predicted promoters (Figure 2). The single closest de novo assembled transcript is 82 bp away from the predicted promoter, while the others are more than 1000 bp away (Table 1). But the absence of transcripts immediately adjacent to promoters cannot confirm the absence of the expression of that promoter, nor can it accurately indicate that the predicted sequences are not promoters. The genome of monocotyledons, particularly rice, is characterized by a large number of mobile elements that can be located between the promoter and the transcription start site (TSS), physically distancing the promoter [19].

The theoretically predicted sequences may also be other regulatory elements of the genome, such as enhancers. Enhancers are small regions of DNA that, when transcription factors bind to them, can increase the level of transcription of a gene or group of genes. Enhancers do not need to be in close proximity to the genes they affect. Recently, it has been shown that enhancers can also perform promoter functions [20]. For example, when a 72 bp tandem repeat from polyomavirus SV40 was inserted into a plasmid without a promoter, this element (an identified enhancer) initiated a low level of transcription, indicating that it can activate RNA Pol II through promoter-like activity [21]. The fact that enhancers can play a promoter role in the genome can significantly complicate the prediction and accurate identification of genome regulatory elements. Consequently, it is necessary to conduct additional studies of the properties of predicted regulatory sequences to determine or confirm their functional role in the genome. For example, epigenetic marks characteristic of known confirmed promoters and enhancers can be studied. Studying the presence epigenetic marks on predicted regulatory sequences can be used to confirm the role of a regulatory sequence as an enhancer or promoter..

The lack of visible transcription behind the promoter region may also be due to weak transcription levels or the silencing of genes due to methylation of their promoter region. Methylation of these regions prevents RNA polymerases and/or various other transcription factors from binding to the promoter region of DNA due to steric hindrance, thereby reducing and even inhibiting gene transcription [22]. The study of methylation status is also an important aspect in understanding the lack of transcription behind potential promoters; it can be studied by analyzing bisulfite genome sequencing data.

## 4. Materials and Methods

### 4.1. Selection of Predicted Promoter Sequences from the Database of Potential Promoter Sequences

In total, 126 potential promoter sequences (Table S1) were selected from the Database of Potential Promoter Sequences (available at: http://victoria.biengi.ac.ru/cgi-bin/dbPPS/index.cgi, accessed on 20 August 2022). This database contains promoter sequences, predicted via the MADHS method, of the following genomes: *Capsicum annuum*, *Homo sapiens*, *Lactuca sativa*, and *Oryza sativa.* We selected the first chromosome of the rice genome. Further selection was performed among the predicted potential promoters located no closer than 1000 bp *upstream* from known genes and no closer than 200 bp from transposons and SINE repeats. This selection was necessary to ensure that the predicted potential promoter is not associated with the transposons and promoters of known genes, and that it is more likely that the predicted promoter is associated with previously unknown genes. Of these, potential sequences were selected with the largest argument of the normal distribution, which shows the degree of non-randomness of the created alignment. The length of the selected predicted potential promoters varies from 428 to 599 nucleotides, and the distance from them to the nearest annotated transcripts varies from 1044 to 92,434 bp.

### 4.2. Analysis of Predicted Potential Promoters for the Presence of TATA-Motifs and Cis-Regulatory Elements

TATA-motifs and cis-regulatory elements were searched for within the predicted sequence of potential promoters. The search was performed by analyzing nucleotide sequences of promoters in the FASTA file using PlantCARE (available at https://bioinformatics.psb.ugent.be/webtools/plantcare/html/, accessed on 4 September 2022), a plant cis-acting regulatory element database [23]. From all motifs found, we extracted elements identified as cis-regulatory elements.

### 4.3. Verification of Selected Sequences

The rice *O. sativa* genome annotation data (available at http://ftp.ensemblgenomes.org/pub/plants/release-52/gff3/oryza_sativa/, accessed on 4 September 2022) was downloaded from the Ensembl Plants integrative resource (available at https://plants.ensembl.org, accessed on 4 September 2022). From these data, annotated transcripts of the first chromosome—6437 transcripts—and sequences of the 5′-untranslated regions of the first chromosome—6474 sequences—were extracted.

The search for annotated transcripts closest to the promoters under study was carried out using Bedtools *closest* [24] with options *-d -iu* to search for transcripts in the downstream direction on the same strand and determine the distance from promoters to transcripts.

### 4.4. Comparative Analysis of Investigated Predicted Promoter Sequences with Predicted Promoters from Another Database

The investigated promoters were compared with promoters from another database, PlantRegMap [25]. This database stores promoter sequences predicted via identification and analysis of transcription factors. A FASTA file with promoter sequences was downloaded from the database (available at http://plantregmap.gao-lab.org/download.php, accessed on 4 September 2022). From the file on promoters extracted from PlantRegMap, sequences and promoter IDs of the first chromosome were extracted using Bioawk (available at https://github.com/lh3/bioawk, accessed on 4 September 2022) and formatted in multi-FASTA format. To obtain the coordinates of these promoters, they were aligned to the genome using bowtie-align-s [26] with options to select sequences that align to only one site in the genome without gaps or nucleotide substitutions: *-s --wrapper basic-0 -a -m1 -v0*. The first chromosome genome (available at https://www.ncbi.nlm.nih.gov/nuccore/NC_029256.1, accessed on 4 September 2022) was downloaded from the NCBI Genome. Then, using the Samtools toolkit [27], the alignment was converted from SAM to BAM format with the Samtools command *view* and options *-b -o*, sorted with command *sort* and option *-o*, and reformatted to BED format with promoter coordinates using bedtools *bamtobed* with option *-i*. Promoters of interest that intersect with promoters from PlantRegMap were found using bedtools *intersect* with the *-s* option and those not intersecting, with options *-s* and *-v*.

### 4.5. Search for Unannotated Transcription Start Sites near Predicted Promoters

To search for possible unannotated transcription behind the predicted promoters, the gene expression cap analysis data (CAGE-seq) of control samples of O. sativa rice (not subjected to targeted stresses, modifications, and infections) were analyzed. Available raw sequencing data were searched for in the NCBI SRA. DRR127211 data were selected for further analysis. Adapters and reads of poor quality were removed using Trim-Galore (available at https://github.com/FelixKrueger/TrimGalore, accessed on 2 November 2022) with options --nextera and --length 51, the quality of reads was assessed in FastQC [28]. Alignment of the filtered data to the genome of the first chromosome of rice O. sativa was performed using Bowtie2 [29] with the -very-sensitive option. The alignment data in SAM format were converted into a BAM format file via the Samtools command—view. Then, alignment data in BAM format were sorted using Samtools sort and were indexed using Samtools index [27]. Furthermore, using the Deeptools [30] command—bamCoverage, the obtained data were converted into peaks. The distances from the mapped 5′-ends of transcripts to the investigated promoters were estimated using the Bedtools [24] command—closest with option -d. Peaks ranging from the intersection with the 3′-end of the potential promoter to 1000 bp in the downstream direction from the 3′ end of the promoter were sampled. Visualization of the selected data was performed using PyGenomeTracks [16].

### 4.6. Search for Possible Unannotated Transcripts Downstream from Potential Promoters

To search potential new transcripts, de novo transcripts were assembled from raw RNA data sequencing available from the NCBI SRA. The following RNA-seq data sets were selected for de novo transcript assembly: SRR11829613, SRR15060372, SRR8469588, SRR11825481, SRR12046251, SRR8146491. They are the results of the RNA sequencing of control samples, deliberately not subjected to stress, infection, or genetic modification. Data loading and conversion from SRA to FASTQ format was performed using the SRA-Toolkit 3.0.2. (available at https://trace.ncbi.nlm.nih.gov/Traces/sra/sra.cgi?view=software, accessed on 15 December 2022 and the SRA Toolkit Development Team) with help of the *fastq-dump* tool. Then, using Trim-galore (available at https://github.com/FelixKrueger/TrimGalore, accessed on 2 November 2022) with options *-illumina -q 28*, Illumina sequencing adapters were removed from the reads in addition to low-quality reads (quality less than 28).

De novo transcript assembly from RNA sequencing data was performed using Trinity (available at GitHub: https://github.com/trinityrnaseq/trinityrnaseq/wiki, accessed on 15 December 2022). To assess the expression level of the assembled transcripts, a method based on the normalization of transcriptome sample data was used, and the number of transcripts per million mapped reads (TPM) was calculated using Salmon (available at GitHub: https://github.com/trinityrnaseq/trinityrnaseq/blob/master/util/align_and_estimate_abundance.pl, accessed on 15 December 2022).

To obtain the coordinates of the assembled transcripts, these transcripts were aligned to the reference genome of the first chromosome of rice *O sativa* using a Bowtie with *-a -m1 -v0 --sam* options, which allows for selecting sequences that are aligned only in one part of the genome without errors. Then, using the Samtools toolkit, the alignment data were converted from the SAM format to a binary file with BAM alignments with the help of the *view* tool, sorted with the help of the *sort* tool and indexed via Samtools *index*. Then, sorted and indexed data were converted to the BED format with the coordinates of the collected transcripts using Bedtools with the *bamtobed* command.

To select potentially “new” transcripts that had not previously been annotated, a search was made for assembled transcripts that did not overlap with those annotated using Bedtools with the *-a -m1 -v0 --sam* options. Information about annotated transcripts was taken from the genome annotation file of the first chromosome of the rice genome (available at http://ftp.ensemblgenomes.org/pub/plants/release-52/gff3/oryza_sativa/, accessed on 20 December 2022) downloaded from the Ensemble Plants Integrative Resource website (available at https://plants.ensemble.org, accessed on 20 December 2022). From these data, annotated transcripts of the first chromosome—6437 transcripts—and sequences of 5′-untranslated regions of the first chromosome—6474 sequences—were extracted. 

### 4.7. Analysis of Potential New Transcripts

To search for potentially novel transcripts, transcripts that are up to 5000 bp away in the 3′ (*downstream*) direction from potential predicted promoters were selected. The selection was made using Bedtools *closest* [24] with options *-iu -D a -s*.

Selected transcripts were checked for the presence of ORF in ORFFinder (available at https://www.ncbi.nlm.nih.gov/orffinder/, accessed on 24 January 2023) with options “ATG and alternative initiation codons” and minimal ORF length (nt) = 150; in cases where no ORFs were found, the minimum length was reduced to 75 nucleotides.

Homologs were searched using the blastx algorithms at NCBI (available at https://blast.ncbi.nlm.nih.gov/Blast.cgi?LINK_LOC=blasthome&PAGE_TYPE=BlastSearch&PROGRAM=blastx, accessed on 24 January 2023) on tomato (taxid:4081), *Arabidopsis* (taxid:3702), and rice (taxid:4530). 

### 4.8. Study of the Degree of Chromatin Accessibility in the Region of Predicted Promoters

Chromatin accessibility was analyzed by examining ATAC-seq sequencing data to recognize open chromatin regions available for transcription factor binding. Data from NCBI SRA (available at https://www.ncbi.nlm.nih.gov/sra, accessed on 10 February 2023)—SRR15311718 were taken for analysis. Data quality analysis was performed in FastQC [28]. Adapters and low-quality reads were removed using Trim-Galore (available at https://github.com/FelixKrueger/TrimGalore, accessed on 2 November 2022) with options *--nextera --paired -q 26 --length 150*. Alignment to the rice genome was performed using bowtie2 [29] with the -*very-sensitive* option. The alignment in SAM-format data were converted in BAM format with the help of the Samtools command—*view*. Then, alignment data in BAM format were sorted and indexed using Samtools with the help of the commands *sort* and *index* [27]. The obtained data were converted into peaks using the Deeptools command [30]—*bamCoverage*. At the next stage, peaks intersecting with predicted promoters were selected using the Bedtools command *intersection* [24]. Visualization of the obtained data was performed using PyGenomeTrack [16].

## 5. Conclusions

In this study, we identified from the 126 predicted promoter sequences of chromosome 1 of the rice genome those sequences that are most likely to be promoters for further experimental validation. Most sequences contain TATA-motifs and some cis-regulatory elements. We identified 14 predicted promoter sequences followed in the downstream direction by unannotated transcription start sites, 5 sequences followed in the downstream direction by potential unannotated transcripts, and 16 sequences that are located at a site in the genome in the open chromatin region for TF seeding. We identified four predicted promoter sequences (Sr. No.: 9, 41, 64, and 81) which we consider the most promising for further experimental analysis.

For in vivo/in vitro testing, constructs with reporter systems such as GUS or GFP behind potential promoter sequences can be used. Y2H/Y1H assays or Bimolecular fluorescence complementation (BiFC) also can be used. But, unfortunately, even such an analysis may not show the real result, as it depends on a number of factors. The tissue specificity of certain classes of spatiotemporal promoters is difficult to properly assess due to the apparent lack of differentially differentiated tissues. Also, the size of the isolated promoter can negatively affect the assay, as the cloning of the promoter may miss deleted essential cis-elements, or may lack the transcription factors necessary for interaction with cis-elements. Additionatly, in in vivo assays, promoter sequences can be randomly integrated into independent genomic events during transformation, which can lead to large expression variability depending on genomic location and may also cause silencing.

Despite the difficulties encountered, the study of genomes, and in particular regulatory sequences, is an important task of modern biology. The genomes of many organisms are already fairly well annotated, but there are still gaps and functionally unknown regions remaining. Knowledge of the exact reference sequence of the genome and its functional components can play an important role in genome editing technology. It is important to know all the areas in which editing can take place, as well as sensibly assessing the possible off-target effects of editing. Precise knowledge of the location of functional elements of the genome will allow us to notice and control random mutations in these areas, which can affect the expression of some genes and have a noticeable or imperceptible effect on the whole, living, edited organism. 

## Figures and Tables

**Table 1:** Unannotated de novo assembled transcripts located not further than 5000 bp downstream from predicted potential promoters.

Potential Promoter Sr. No.	Distances from Potential Promoters to De Novo Transcripts, bp	Length of De Novo Assembled Transcripts, bp	TPM	Number of Reads	ORF Lengths of De Novo Transcripts (Nucleotides/ Amino Acids)
5	4991	227	21.705628	10	111/36
36	4141	220	19.372170	8	129/42
37	3653	638	5.662674	77	465/154
70	82	414	10.787241	62	366/121
124	4633	860	44.718849	956	396/131

**Table 2:** Proteins from *A. thaliana* homologous to de novo assembled unannotated transcripts located behind predicted potential promoters in the first chromosome of the rice genome.

Potential Promoter Sr. No.	Proteins from *A. thaliana* Homologous to De Novo Transcripts of *O. sativa*	Query Cover, %	Identities, %
37	Molecule 5MDX, chain D	69	96
37	Molecule 7OUI, chain A	65	20
124	Molecule 6KKS, chain A	25	63

## Data Availability

The data presented in this study are available on request from the corresponding author.

## Supplementary Materials

The following supporting information can be downloaded at: https://www.mdpi.com/article/10.3390/plants12203573/s1, Table S1: Predicted potential promoters from 1st chromosome of the rice genome: the coordinates of the beginning and end of the predicted promoter sequences (start and end) in the *O. sativa* rice genome; DNA strand orientation (strand); the length of promoter sequence (length) serial number of predicted promoter (№); nucleotide sequence (sequence). TATA-motifs are in bold.
