# Supplementary material for: In Silico Verification of Predicted Potential Promoter Sequences in the Rice (Oryza sativa) Genome
Source: Plants (Basel). 2023 Oct 14;12(20):3573. doi: 10.3390/plants12203573 (PMC10609952; doi:10.3390/plants12203573)
Supplement: Supplementary file 1 [file plants-12-03573-s001.zip › plants-2634331-supplementary.pdf]

Table S1. Predicted potential promoters from 1st chromosome of the rice genome: the coordinates of the beginning and end of the predicted promoter sequences (start and end) in the *O. sativa* rice genome; DNA strand orientation (strand); the length of promoter sequence (length) serial number of predicted promoter (№); nucleotide sequence (sequence). TATA-motifs are in **bold**.

Cis-acting element involved in:

- the abscisic acid responsiveness (ACGTG, AAACA, CACGTG, CGTACGTGCA),
- low-temperature responsiveness (CCGAAA),
- the MeJA-responsiveness (TGACG, CGTCA),
- anaerobic induction (AAACCA),
- gibberellin-responsiveness (TATCCCA),
- phytochrome down-regulation expression (TGATTTTACA, GGATTTTAAAGT),
- light responsiveness (CACGAC, ACACGTGT, TACGTG, CACGTT, CACGTC),
- auxin responsiveness (GGTCCAT),
- meristem expression (GCCACT).

As well as just: учтено

- cis-acting regulatory element (CCGTCC),
- common cis-acting element in promoter and enhancer regions (CAAAT, CCAAT, CCCAATTT, TGCCAAC, CAACCAACTCC).

(when crossing different cis-acting elements, some parts were highlighted by changing font color)

Sr. No.	start	end	length	strand	sequence
1	5634017	5634492	476	-	GATAGGATATTTTGTCCCCAAAACAAAGTTGGGTTTGTAAGCTGCTTTGGTTACCTAAAATT<br>TTGCAACATTTTTGTCGCAATTTTTGTTGT**TATATAT**GATTTTTCTTTATTT**TATATAA**GTTT<br>GAATATTTATGCATGTAACTT**TATA**AATTT**TATA**TGGACTGTAAGTTTATTTCATGTTTAAG<br>TTAAAATAATTTATTGTTCAATCCGAAAGAAGTAACTTTTACAGTGGAGCGATGAGAAGTA<br>TTG**TATA**TTGTCATTCCATGAATGCAAAAAGTGACTGAAA**TATTTAAAA**TTTTATGGACCC<br>GAAATCTTTACATCCC**TATA**AATTAAAATGTCTCCCGTGACGTGAGCAAAAAATTCA**ATAT**<br>**AA**AACAACTATTACAACAAAAGCATGCAACTT**TATATATA**CATGTATTTCC**TATA**CAATAC<br>TCCCTTTATTTTTAA**TATA**CGACATTGTTTTATCTCATTAAAAAA
2	23193444	23193966	523	-	GAATCTGGTGCTATGGAGAATGGAGAAAGAATCTAGAATCAGGTTGTTGTTTTGTTGACTT<br>GTTTTCCCTTTATGAATTATCCTCATCAAAATAAGTGCATGTAACAAAAAAAAAAGTT**TATAT**<br>**AA**ATAACATGATGTAACTGATGACTTTAACATTAACAAACAAAGAGGTGTAAAAGTAGTAA<br>**TATA**CATGTGTTCAGATAACAAGAATTTGGTAGAATTTACGATCCCATGATTTTC**TATATT**<br>**TATATTT**CAGAAACAACGTTTAGAATTAGCACAGAAGTATTGCATGCTTCACATAAACCAT<br>CACTTCATTTAAGGACAAAGGGCCAAACAAAAAAGATGTGCAGCAAAGCCAACCTTTCGTG<br>GAGTACTTTTGATAGCCTCCATAAAACTTTGCATCTCCCTCAAATATGACAAGGCTATCTGA<br>TAATGGTGTTCATTCCCATCAATTGCACTCTTTTCCATCTCTCCTAGAGAAAGGGCGGCTGA<br>CTGAAGCGCATATCGCATGTGCATCAGCTCAA
3	24122935	24123517	583	+	AACAGCTTCTAAATGTCTAATCTCCCCTCTCTAACCTCC**TACAAAA**CCATTTGCCTCTTG**TA**<br>**TA**TCACTGCTCAGTGCTCACCTTGTACCAAGCCGTTTTTCAAGAACATTTGCGAATTGTTAG<br>TCGTTGGCTCACTTCATTATGCATATCTTGTTTGAATCTTTGTTTCATTGTTTGGTCAGCCGG<br>TCGAGTTAGCCAGCAACCATTGCCAAGAGATCAGTGAAACCTATCCTCAATTGGTTCCTGA<br>AATGCTGGCCATGGCGCCATGTCGATTATGTCAGATTGGTCAGTGATCCGGTCAGGTTAGG<br>TCAGAAGATCTCCGGGTTGCCGGGGCCGTGGGGCATGGCGCCGCCGCCACCGCCGCCGCG<br>GCGCAGGTCGGCCTGCCGGAGGTGCAGGGACAGCGCGTAGTTGGACGCCTCCAGCCGCC<br>GCACCTGCTCCCTGTAGTCGTCGAGCTGCCACCGGAGCTCCCGCTGCGCCTTCTCCTGCGCG<br>GTCCGCAGCCGGTGGTGGAACAGCACCGCCCTCTTGAGGAACCCGATCTGCCTCGACGCG<br>GCGTCCAGCCTCGCGGCGGTGCCAGCGCCGACA
4	24237415	24237903	489	-	AATTC**TATA**GCACCTCCGGTTACGAGGGACACGGTCGCATGCTCTGTAAAGCTTGGAATGT<br>ATTTTTGATGGAACCACTCAGCTTCCTGGAGGTAATTGTTGGATAATATTTGAAACTGCATG<br>TGGCAAAAAAAACAGAG**TATA**ATTAAGATAAATACATATTTTTGAAGATGTATGTATTAAT<br>TCTTAAAGTTGCTAGTTCAGATTTT**TATA**CGTAGGAGTTGATTGAAAAAACAATTTCTTTTT<br>TGTCCATTTCTGTTATGGACGATTATCTATTTATCCATTTCTAA**ATTATA**AGCTGTACATAAC<br>TTTGTTGAAAAGTTG**TATA**CGACGCTTCGTACCGCTTTAATGTTGTAAGCATTACGATATTT<br>CTCATGTGGTTCCAACTCATCTTCAAAGTCCCTAAAGTTGCTCATAATACTGAGAAAGAACC<br>TCTTCAGCCCCTCCGGTACAAGTGAAACAGCATTCTTATCCCATCTGCATTGTCAA

5	26303795	26304223	429	-	TTTTCTTC**ATATATATATATA**GCTCACACTATT**TATA**GAGTTTGGTGCTTAATTATTGTATT<br>AAATGCTTACTCCAATTACAGCAAGTGATGTTTTAGAATTTGTAACATAATTTAATTTCAACT<br>ATGATTATCGTTTGGAA**TATA**GGTTTTAGGCATATGAAAATGACATGGCCAACTTTGT**TAT**<br>**A**ACAAAAACTTTCGTAGTATCGCATTCATGCAAAAGAATTATTCTGTCCAGACATAAACATT<br>TATGATAATATTTGATTATTTGGTCAAATTTGTGAAAATTGACCATCCATAATTTCTAAAATG<br>TCTTTTTTTTCTTAAAAAAAATGTTATG**TATA**GATTTGCCTTGAAAAATAATGTCATAATAGCC<br>TGAACTTAGATTT**TATA**AACTTATTCTTGTAAAAATTGATGGCCAAAGTTTCCAA
6	26777727	26778264	538	-	TTTGTCTTAGATAATACTCTTTAGATCTAATTTTGACAGCTAAATATGTTTCATTTTTTTC**TAT**<br>**A**CGACTGAACTAGTTGTAGCAACCTTTAGTTTTAGGTTTAATTTTAA**ATATAA**TTCATCACA<br>ATCTAATTAAATAAGATTAAAAAAAGATATTTTTAACAGTTTATGCACCCATCTGGAATGAT<br>GTGATCTATTATGCTCAACAACATCTTTATTTTGATTAC**TATATAA**ATAATTGAAATAATTA<br>GACTAAGAAGCTGAATTGCT**TATATATA**CAATTAATTATGTATGAAAACAAATATTTTTTTT<br>TATTAATATGTGGATTAGAAAACTCAATTGTTTTCTTTAATAAGTTTGTATCGTTGAGTTGTG<br>GCTTAAAAAATCTCGAGACAGTCATCAAACCGATTACCTATTTTGTTTCAATCTCCTATTCAT<br>CTTGTCTGTTTGCACCGCGTCGATTAAA**ATATAA**GTCAAAACTAAAACTTTTAAAAAATAA<br>TCATATGCCCTCTTCTTCCTCATAATACCAATGGCTTTG
7	28002899	28003459	561	-	TTGTAGGTGTTTATGTTTCGATGTTGTAAATTACTTTAGAATTTTTGTCGGTTTTAGATTTTA<br>CGTTTGGAGCACGGTAACACTAGATGTTTTGTTTGTCTTCACACCTCACTTTCTCTTTTTTTTC<br>C**ATTATA**TCAAATTTG**TATTTAAA**ATTCTTTTAGTGTAGTAGGGTTTTATGTTTTGATGTT<br>GTAAATTACTTTAGAATTTTTGCTGGTTTAAG**ATTATA**TTTTACATTTGGAGCACGGTAACA<br>CTAGATATTTTGTATGTCTTTCCACCTCACTTTCTCCATTTTGTCCATTCTATCAAATTTG**TAT**<br>**TTAAA**ATTTCTTTAGCGTTGTATGGGCGTATGTTTTGATGTTGTAAATTACTTTAGATTTTTT<br>TTTGCCGGTTTAAGATTTTACACTTTGGAGCACGGTAACACTAGTTATTTTGTTTGTCTTCCC<br>ACCTCAGCCCTTTGGATCATCCATCAAAGATTTTTCCTGTTTCCAACCCCCCCCCCCAGCCCC<br>CTAAAAAAAGAAAAAAAAGAAAAAAAACAAACATTAAACAAGAAGTGATAGCTCTGAA
8	28409073	28409656	584	-	AAACAAATACGATCACGAAAAATTCTTGCAAGGTGAAAACATTTCGAACCCAGCTCCCAAA<br>ATCAACGAGTATCATCTACTTGATACAATTTAGGTCAGAGCTAATCTGCTAAATGTACATAG<br>AGCCTGAAGATTATGGGGGTATCGGTTTGTAGCGTTACCGGGGATTAGAGAAACTAATCTT<br>CAGGAGTGATTTTACAGTTTTT**TATA**AGTACTAGGAGGTATCATATTTTATTGTAGCGTAAA<br>ATTTGGCATATCTTGATACCTCCGATACCAGGTCATATGAAAATTTACACAATTTTGATATCT<br>TTCGGTATCTCTAAAATCTGTAAAATATCTCAATCTTCAGTTAACTGTGAACACCACATGGA<br>TCACACAATTCAAAGCGACATAAGAGGTCGGCTGAGCCCGGAAGAGTGAATGAATGACAG<br>AAAAGTGTTGAGCTTAGCGGCAGCGGTCCCGGTTTCGTAAGACCGGGATCAAGAAAAACG<br>ACTTTTTTTTTTTACCCCATCAAGCGGTTGCAATCACGCCAGAGAGCGCGCCCCTCCCGGCC<br>TTCTCCTCGCCGCCGTCGTGTTCGTCATGGC
9	28832481	28833057	577	+	**ATATAT**CTTCCTTGACCCTTTTTTGTTTTACAATTGAGGGTTGTAGTAGTATTAATTATTTGG<br>ATTTTAAAGTGGGATTTGAGAAGGTGCTCTCTGTATGTATCTACGCGGTGTCGGTTTATTAA<br>GCAACGAGAAAGAAATATTGTCCTACAGATGCTGATATGTGGGATTGTCCATGTTCGATGT<br>GCATGACTGCAAATGAGCTCATTTTTGTGCTGTACGTT**TATA**CCTATGTGGTTATCCCAAGA<br>TTACCG**TATA**TGATATGTAAATTCTGCACATACATTTTCATATGCAGCAATTCAAATGCTCT<br>ACCTATGTCATTATCGGGAATTAATTAACCAGATGGGGAGAAGCAAAACATACCATGATAC<br>CGACGCAACTGAAGATCGTGTTTGTTGCCGTTGGAGGATCAGTGAGGCACACGCACGCGC<br>CGGGATCCATACCGCCAACGGCGCCACCGCGCGAGCAGAGCGGAGATCTCGACGAATCCC<br>CCCCGTACAGCAAGCCCAGCCACGGCCGCCGGCGACGCTCCCCGAGTTCCGAAGGCCGCT<br>CGCCGCCACCGCGACAACCGCGTGGT
10	29559166	29559731	566	+	ATCCCATCAGGGATGGAAAACAATTGATTCTATTAATGCTTTCCTTCAGTGGCAATGACTTT<br>TTCGTAGAATAGCTTGCTTCTTAAACCGCGCTATTGTGGTCTGGTTGAATTTTGCTCCTTTTA<br>CTTTGTAGCCAAGGGAGGAACTCAAACGGAAAGAGAAAGATCACCTACAAGTAAGGCTGG<br>CCTGACCATAGTTGTACTAAAAAAAATGCACTTC**TATA**CGCAGTATTGCCAATCAAATTTAG<br>CAAAGAAAAACGGTTTTTCTATGTACGAGCAACTGAGCAGCCGGAAGGAGGTTTGGTGGA<br>ACAGCGACACCCGGCCTCCCACCCCACGGGTTCCGAGAGATTCTTTGGCGGTAGGTGATCC<br>CAACCAACGTCGTCCAGGCCCCACCCCGCAGATCCACCAAACCACACACGGCCGTCGCTGA<br>CTCACCGATGCACCATCGCCAACCGTACTCAGCGACCCCGCGCGTCAGTGGGCGGATCGG<br>ACCGACGAGCCGCGCTGCTGGGTGCACTGTGCAGCGCCCACCCCCGCGACCCACGTGTTCC<br>CGTCCCCGCCCATGCT
11	29561875	29562462	588	-	TTTAATCTTAACACCGCAAGAAATCAGTGAAGAAAAGCAGCATGCCTTTTAACATCACATAC<br>ATGTTAAAGATCGGGTGTGTTAAGTAGGGGCTCTTGCGCACAAGGCTACTCGGAATTGAG<br>CTTGACACCTCTGATGCGAGGGATTCAGCAGTGAAGTTCACCTAAAAATG**ATTATA**ACGA

					CACGATGTTTTAATATCATGATCCAATTTCACATCAAGCACACAAATTTTGCGCCGATTGAA<br>TGATTACTTACTGGCTTGCCACCATTGAACACCTTGAACAGCTTGTCGACATCCTCTAAGGT<br>TGTGGTCTCATCAAAGGCCACGGTTATCTGCAACATCACAGCTTGAATTTAATGCTCAACAC<br>TCAACATCAACTAACTCGGTTGCAATAGAACGGAAAATGTCAACAGAAAGCGAACCGTGG<br>TTGCATCAACAACACGAAGGTTCATCTCGTTCTTGCAGGCCTCCTGGGCAATCGCATTCGCA<br>TCAGCGACCTTGACCTTGACAGTGTCAAAGAACGGCAGTTCCTGCACAGTCACTGTTCCGA<br>GCTTCTTCAGTCCATGGGCAAAGGTACCAGCCAGGCC
12	29593372	29593947	576	+	ATATTGAATTATTGATGACTCGCTCTCGTGTTCAAGACGACCCCAAGCGCCGATCAAAATCT<br>GTTTTTTTTTTTTTGTGCTGATGATCTTCCATCTCGTTTCTATTTGAGATAC**ATATAT**GTCTTC<br>CCTTCTGTCGTATGTCTCGTACGAACGTATCGTATCCTACCCAAAATCCTGTTCTAACTGGTA<br>AGTCCGGCACTTGCATGAATACATCTCTTCGGTTCGGGCGTTCGGCCCTTTTATCATGCGTG<br>AAACAGAGTCTGCGCGTAGGCACGCCAAAATCCCTGTCAAGATCTC**TATA**GCTTCCACGCT<br>CCCATCAAGCTGAATCGATCTCCGACGAAGAAATTAGCCGGACATGGCGAGAACAGCCCT<br>GTACGGCAGCAAAGAGGTGGACCCCTTGTTGGTGCTACAGACCAAAGTCATAATCCTGTTC<br>TATGGTATGGTGACGATAGTTTTAGTGCTGGGGATCCCGGCCGCTGCCCTTCTTTCTAACGC<br>CGCCGAGAAGGTCAAGTACACCTTGGATCTCGCCGCCGTCGAGGGGATGGACGTCGCCGC<br>CGCCGCCACGGCCGGCGCCGG
13	29594054	29594586	533	-	TACCAAAGTTAAGTGACGACTTTCGATGCAACAAAATTAAAAAACGAAAACCTTCAATGC**T**<br>**ATA**AATCAAATTGTTATTACTCCATATCCTTACCAAGGAATAATGTTTTCCATTCTATTTACT<br>TTGGAGATAACATGCTAAAAATATGAATTA**ATATATA**TGTTCCTATTTGGAAAAAGGAAA<br>AAAAGTTATGATACCAAACAAGAAAACGTGAACAACGAAGAAACAACTATCCATCAGCCTT<br>CCCTTGACCAAACTGTTATTCATCTCTTCCTCCCAGCGTAAGCTCCTGTGAAATCCCCACCAT<br>CCCAGTGCTAGGCGCGAAGGCAGCCATACCGAAATTAGCGTAGTAGTGCAGCTTCATCTCG<br>ACGTAGACCTTAGCCGTCCCCTCCTGCAGCTCTGAGACAAAGCGTTCGCGTAGCTCCTGCG<br>ACAAGTGGACGTTCTTTCCCCAGGGCACCACCGTCAGCTTGGCCGTCGACCTCTTCTGCACG<br>CAGAATCCCGGGACGCGCCCCCACGCGAGCGCTACGCCGGA
14	29655728	29656263	536	-	GTGCTGCTGCTGAGAGGCAGACTACAATAGGATCACCACTAACCATTTATTTTATCTGTTAT<br>TTAAGAAATGTTTATTGCTTTACAACACTAATTTCTTATCTGAAACCAGGCTTCAAGTTGATG<br>CCATTTCAAGATTGTCTTCCTTTGTTATGGAAAAACAAGAAAGTGATGCGGCCAAGAGAGC<br>TCTTACTGAAGCTCGTGAAAGAAACGAAGATCTACTGAAGAGAAATGAGGACCTCCTCAAA<br>CGGAATGATGACTTGATTAAGAAAATTGAAGAGTCTAGCAAAACTATTACTCAACTTCAAG<br>AGACCTTACAAAGGTGCATATGCAATTTTTATTTCTTATTTTTACCTATTTGTCATGATGGTA<br>TCAGGCTCACTCTATCATTGTCAACTTCAATTAAATAACTCTTTTCCATCCTTCCGTAAACAG<br>GTTGGAAGGAAAATCAACGAACTTAGAAGCTGAGAACCAAGTTCTCCGTCAACAAGCAAC<br>TGCAACTCCACCATCAACAGCCAAATCTTCAGCTTCACGCTC
15	29682929	29683518	590	-	CATTTTTAAAATTCGAATACATGAATCCCTGAATTCAATCTACCTACACAAATTTTTGACAGG<br>TACATCTTTCCACCCCAATCTGTCAAGTTCAGGAAGGGAATGTATCAATCGCATGAACAAG<br>AACAACCCATGTCATCTTCTGCCCCTTCCCAAGTTCCAAACCCTTCAATACGACCCCAACATC<br>CATCCACCAAACCTTCTCCTCCGCGAGTTCCACGACTTCTTCCATGGGTTTCCACTCCACCAC<br>GAGACAAGAATTCTGTCCTCCTCAGATCCCAGATCCAGCTGACTGAACAAGGCCAAAGCAC<br>CGAGCCGGCGAGCCCGCCGCACAGTAGCTGCAGCAACACGACGCAGGAAACCAGCGGGG<br>CAAGGCAACCAAAGCCAACAGCCAAGCAAGGCATCCGCTCCGCTCCGCTGCCCCCTCACGC<br>CGGCCGCGCCCTCCCTCCCCCAAAAATCCCCACCCCAAAATTCCAAAACTCAAACCCTCCTCT<br>GCTGCTATCCGTCCATGTCGTCCCGCTACCTCGTGCCGCCGCCGGGGGCCGCCGACCGGGC<br>GGGGGGCGACGCCGGGGACGCGGCCCTCGCGCGCG
16	29854966	29855558	593	-	ATATTTTTGGCCGTCTGTGCTTTTGCTTCTTCCAGAAAGCGAGCAACCGTTCGTTGGCTGAT<br>GATGGTCCATTTTTCAGTGTTATTCGGCCCCAGCTCTGGTTCTTGTTCTATGTTTGTGATTAT<br>GTTCTCTGGTCCTGTTTCTGAATTTGTTCCTTTTTCTCTCTAAGAACATCTTTGTGAAAGTTTT<br>CGATGTAGTAGATGTTACAAGGCGATGTCACTGTGCATGTCCAAGTTTATGTCGCTTTCGTG<br>ACATTAGAGCACGTACAATGCCTAGCCCTAGGTCATTCCCCTGGCTCACGCCATCGGCCTCC<br>ATTGCCTTTCGTGCGTTTGCTCGCGGGGGACGCGCCCTTCGCCCACTTTTGGCTAAGCCACG<br>GAGCCGAGCTCTGTGTTGGGCGCACAGTGGCTACCAATGTAGCTCATGGTCACTGCTCCCA<br>CCCCAAAATTTCCCAAACGACTCCTCGGGACTCTCCCACTCTCGCCCCTCGTTCTCATCGACC<br>TCCTCGGCGGCCGCCTCGCCTTCTCACTTCACCGGCCGCAATCGTCGTCGTCCCCGTGAGAG<br>CAAGCTTCGGGGAGGGCAGATCCACCGTCAAA
17	29915311	29915908	598	+	GCAGAGAAATCAATTATGAAAGAGCTAGTAGTACTCCCCAATTTCTTTTCGTCAAAAAAGA<br>AAATAATAAATCTGCACACGTGTGTGTAAGAGCTACTCGTCGCTTAACAGCGGATGATCTT<br>TCATGACTTGATAGATGGTTGTTGTTAATTTGTTGACTGTGCATTGTGCTAGCATGATTGGA<br>ATGTTCAAGATGAACCTAGCTAACTTGATAAATAAATGGTGGCCACTAACAGTACATGAAG

					GTGAAATGAGAACGATGCACCATAATTTTTCCCCGAAAAATGTTCCTTTTCTTTTCACGAAG<br>ATATGCAATCGTTTGTGCACAATACTAGTACTACTAGCACTATTTACCTGCCCGGATACTGC<br>GTCACTCACCATGCGGCATACCAGTTTTTGGACGGAACGGCGTTGCCTGCCGCGCGAGCGT<br>GGCGTGGCGCCGGGGTCCCCATGGCGAGTTGGCTTGGCCCGGCCGCACGGGGGGAGCAT<br>TAAACCCCCGTTGGCCTCTTCCGCCCCGAAATCCCGTTGCCCCCGACGGAAATCCGGCAAG<br>GTAGAGCCCGCGGAGTGCGACCGTGACGGCGACCGCGACCACGTGACC
18	29924963	29925542	580	-	GTGAAATTTAAATTGGCGAAGATAACAGAAGTGTGGAGAGTGACAATGACACGCCATAGC<br>AAGGAAGTTCCATGCAATGGAGGTGATGAGATGGGGAGGTAAAACGCAAAACTGAAAAA<br>GAAAAGGCAAAACATATCTAGCAGGAAGAATTTTAACGGAAACACGC**TATATAA**GGGAC<br>ATTAATAGGTGATGACGATGGCGAAC**TATA**TCTAGGGATGGGTAAAAATTGAGACAAAAA<br>TCAAAACTGAAAACCCGAGACCAAACCTGAAAAAACATAGTATCGATCATTGGTCCCTAAC<br>TCGGTCTAGGTCTCATGTTTACAGGACCAATTATTTGATTAGTTTATTGCATCGGATACTTG<br>AATTACCGATCTATTCAAGCCTTATCCTAATCCCCTCCTCATTCATCA**ATATAT**CCCTTCCAC<br>TTCCTCATCCACGATAGCACTTGACGCCTCGTATGTTATCTTGGCGCTCCTCTCCCCTCTCCT<br>CACCTCAGAACTTCTTGAGTGGACCTCTCTGGCTAGAATGTCTCCATGGCTTTTGTCCCTCAT<br>TGGCCACCGCTGCCTCAACGGCAAGGTGCT
19	30206231	30206772	542	-	AGTTCATTGCTGAATGGGTTTGAGAATTTAATAATTTGTTTGCAACGGAAGAATTTGAAGG<br>AATTATGAAGGATTTGCGTTGTTTTTTTTTTTTTGCTAC**TACAAAA**AGTATCAGTATTGCAAA<br>TTGGCAACGCCATTCAAATGGTTGAGATGTGTTCAAGAAAGGGTATCTGACATGATGGACA<br>CGCATTTGTGTGTGCAAGACTGATGAACATATGAAGTAATTTGGTTTGTTAGGATGACTTCC<br>AAGTCAGGGAAAAAGGAGGCGGCATCTTTTGGCTTTTGGACCATACTTATCACAGTCCTGT<br>GACG**ATATATA**CAAAGGAACAAACATACCATACAAGAAAATAATGTTTGTTTCGAAAAAA<br>GACCAATCGTTTTCTGGAAATGGAGAAGAAAAGAAAAAGGAATCATAAAAGCGAAGGAC<br>GGGTTATCTCTAAAGCACATATGTACGTGCCACAGCGTCCACCCACGCACAAAGGAAGAAG<br>GCGAGATTCTTCGTCTCAACGAGAAAGCTGATGTCTACACAAGAACTAAGCCAGA
20	30556057	30556605	549	-	ATGTCACATAAGGAAGAGGTAAACTCG**ATATAT**TCGATCACACTAACAATGAGTTGACTG<br>CAAATAGGTACAAAGAGAGC**TATA**GGACAGAATAGCATAATTTATTAAGCAAAAGAACTG<br>GAGTTTCGTAGTGCACAGGACCTTCATCTAATGCACTAAACTAAACATACAATTCCCAAGCC<br>CTAAAGTCCTAATTTTCATGTATTATTCCTTCCATCCTGAACTCATGAAATAAAATGGGAAA<br>ATTTCTAGCATGCTCTCGAGTTTTACTCAATCCCTTCCATGTTCCTAAGAAATGTGTGGTCCC<br>CTCCATGTCAATTCCATCACCTCCTCATCGTTAACCTCC**ATTATA**CTTTTCAATATGGCCAAA<br>TTGCCCATGGCACAGTGCACTTGGCTGACGTCCAAGAAAACAAAAGGGAACAAGAGAAAG<br>AGAGGAAAGCCCATGAGGAAGAAGAAACACCTCACTGATCACTCTTAGGTGATAGAATAG<br>GCAAGCAAATAAGCTTGCCAGTCAGAACTCAGCACCTTAAACCTAAAGGCATAACGATT
21	30557486	30558071	586	-	ATGGGGGGAAATAATCAACAATAAAAGAGTATCATTGCTTGAGGTT**TACAAAA**CAAATCA<br>CATAAACTATTGACGTAATAAGTGATAATAATTTTTGGATCAGCAGATCAATTATTTTTTAT<br>GG**TATA**CACAAAATATTGGGTATTTGTGCATGTATTAAGGAACTTGAGTTTAGAGAAAAC<br>AACCAACTGGATGAAGATCAACCAAGAAAAAGGGGAAAAAAGGGAAGAAGATAATCCA<br>AACTGAAAGTCTCACATTTTTTTTAGACCAGGAAAAAGAAACACCACTAGGTAAACTCCTG<br>GATTGTTGCTCCTGTCAACCTACAATGCCGCTTCCTCCGCATAGATAGTTGGCACCAGGGCT<br>TGCTGAGTTGGTTGAAGTATTTCTCTCCTAAATCATCCAAGTCACCACTCAATGGTATCAAA<br>GCCGAGCTGCAGATGCTTCAGAATCTACCTCTCGCCAAGAGCCGCCAACCTCAAAATTGTT<br>CTAACTGCTTGAGAAGCAGCTTGTCTGTACTCAGGTTAAAGGCACTTAGAATTAACCACCG<br>AACCTGCAGGGATTCCGGGAAAGACTAAGATGTGGCCG
22	30810940	30811525	586	+	AGGGAAGTGAGGTTGTTCTTTTCTTTTCAGTACG**TATA**GACAAGCAGTGACGAAACCAAG<br>ATCTTCGTACGCCTAGGGCCCAAATTAAGCTAAAACTTGCCATGACAAATGTGCGGCAAAG<br>**TATA**GTACAGGATGGAAAAAAAACAAGAGTAAACTAAAATTAGGCAACAACCACATTTGG<br>TTTAAGCACAATACAATCTTGCTCATTGTTACTAGCCATGGTTTCTACTGAATTAGGCACTG<br>GGATGGAGTCATTGGTTTCTTCCGTTTCCTAAGTTGCCGGGGTGGATTCATTTGAGGACTG<br>AAC**TATA**TTGTCCTACTTCTCTTCATCTACTAATTCAAACCAAATCTAATTGAGTAATATGAT<br>GTACTAAAGC**TATA**GGTTTGAGATTTCAGATTTGAGAAGTGAGAAAGGAAGAAGAAAGA<br>GAACAAAAAATTATTGACCTTCCACTCCCGCACGACCGCCACTGCCACCGCACGCCCGCAC<br>GGACCGCACGCCTGTTGCCTGCAGGCTGTCGCCTCGTGCAGCTGCGCCCCCGCTGCCGCAC<br>GCCGCCTCGCACCTCCCCGCCGGCCGCCACCCTATCGA
23	31133856	31134440	585	-	GCACTG**TATA**TTGTTCTTGTAACCATTTGTTTCAAGGGTATGACGGTCTGGCCGGGCAGCT<br>ATTGGAACAACCATCTTAATCACGCCTTGGTACTTACTGACCCTCTAAGGGCAATGCCAGTT<br>TTAAGAGACATCTACATAGAAAAGTTTGCAATGAACCCTGCAACCTACCGTCTGGAAATAT<br>GTTAAACTATCAAATCAATGTCGCTGTATGATAGCAAGAACACCTTCTCTGTTAAGGCGCCT

					GATTAATCTTAGAGCATGTGCATTGTTGAATTTAGAGAGGGTTC**TATA**GGAGAGAGAGAA<br>GGTGATAAGGCCGATTCTACGCATTGCAAGATTCTCCCTGTTGAATTTAAGCATAGTGACA<br>GGCATCAGTGCTAGTGTAGAAAAAAG**TATA**TTGGACCCGCATGTCAGATATCATTGCTCCC<br>AGAAATAAAGTGGCCAATCTCTATTTCCTTTTTTATTCACGTGGGTCCCAATTAAAGAACAC<br>GTGGGTCCCAATTTGATCGGCCTGAGCTTCGCCACCTCAGCGCCAAGGCGTTTGGTGCTGA<br>CCTTCGGCTCTAGTACACCTTCACCGCGAGGGGG
24	31415775	31416350	576	-	GAGAAAACAATGATAGGATGCCGGTAGGAGAGGAGGGCGCTGGCAGGATAGTGTTGACG<br>ACGACGAGAGGATGCCGGCGGGAGAGGCGGTCGGCGCGAAGACATCACCGGCGACGAG<br>AGGCGGAGGGAATAAGTAGGAAGGAGGTGGTGGCGGCTAGAAGGGGCGAGGCAGCGA<br>GAGCAGATGGATCTTCATCTATGGGCGGCAGCGACGGAGCTTCATTGCCGCGAGCACCCC<br>GTTGAGACCAACGGCGCCCCACAGCCGACCCTCGGCCTACTTTGGCTTCGTCGTCGACGCC<br>GCCCCCACATGCCACCCTAGATCTGCCTTGCCTGGACCACCTAGGGACCTTCACCACCACCT<br>CATCAACCACCCCACCGTCGGCACAGCCCTCCACTGACCCCGTTGCCTAGCAGCCAACCCAC<br>CTCCGTCGTCTCCATCTCAGACTCCTCCCGGCGCGAGCTCGCGCTGGCCTCGGCCCTCCACT<br>GTCGGCCGCTCCACTCCGCCGCTCTCTCGGCCCTCCTCTCTCGCCAGCGTGGCCGGCCGCCT<br>CTCCCCTCGCCAGCTGCTCTGCCCACCGCCGCC
25	32105302	32105851	550	-	GGCAAGCAGCTCACTGAAGCATGATAGGAAGATAAACATAATCTAGCTAAGCATCATCTTA<br>TTGTTGTACGTACGTGCAAGTGTAGC**ATTATA**TTGCCTAGTCACAAATAAATTAAGAACTT<br>AATTTAGAAGCTAGTAGCAAAGTAAATTCGAAGAGGATGCTGAAGTACCCAGGAAAAAG**T**<br>**ATA**TTGAGGCTAGGAAAGGATCAGCACAACGTTGCAATTAATTGTGAAATTGTAAAAATG<br>CAGCAAAATGATTTTCGATCCATTTATTAATTAGGTCAAAGATACG**TATA**ACCGTACCAACT<br>GATCAAATTGATGATCAGATTGACATGTGAAAAAGGAGGATCAATAA**ATTATA**CTCCTAG<br>**TATA**TGTATTGGGGC**ATATAT**GTTTTCACTTGATTGATAAGCCGGCTAATCC**TATATA**GC<br>TTAAGAAATCATCAATTACTATGTTTTCTCATACGTATTATTGTCAAATTA**ATATATA**TGTC<br>AATTAAGTTCCCAAATTAACTTTCTCAGTCAAATTGATCTGCTCCTCACTAATTAATTTGCCA<br>A
26	32194446	32195041	596	-	AGGGGTCGTTATTTGATTATTTTCGTAGGCACCGAAAAA**ATATATTATA**TTTAGTATTCAT<br>GGAAGCTTAAATAGGACAAACTATGCATATGTGGCTGAATATCATTTTGGAGAGTACGTGT<br>TCCTGATCCATAACTCC**ATATAT**GCTACATTGTGCTTAACTTCTGCCTCCAATTCCTACTCTT<br>GCTGAATTTTCCTCCTGCAAAGTGCAAACAGGCAGGAGATCGTATTAGTTGCTAATATGAA<br>ATTAACAAAACTTCTAATTTCATGCAAGCCGTCGCTATGCCAATTACTCATCTCTGACTGGAT<br>TACGAGATTAATTAACCTCGTTCTTTTCAGTCAAAGTCTACAATTTCCAAAACAAAATCAACA<br>CACAAACAAATTGTCAAAGACTCAGACCTTGAAATAAACGACTGCTTATCTCTGGTCAAGA<br>AATCGCCGGAATAACAAACAAACAAACAACCGGGAAA**ATATATAA**ACAATAAAAGGGAG<br>AATGCCGATCGAGCTAGCTCGTGCATTCCGGTCCAAAACCGTTGGCTGCTCGACCGAATCA<br>CCTACCGTGAACCAGCCGTCTTCTTTACCTTCAAGCCATCTCC
27	32402570	32403152	583	-	AAAAGCATATTTGTCCGTACAATGCATGGCTCCCCTTAGTTTTGT**ATTATA**TGATATGAAA<br>AAAAAAACAATCGGGTAAAAAGAATAGGAAAAAAAGAACCATTAGTTTCAT**ATTATA**TGA<br>**TATA**GATACCCTTTTAAAATAATATTATTCTGCTGAATAAAAATGAAGGGAGAGCTAGCAG<br>AGGGACACAGTCATTAGGGGATCACGTTAATCACATAAGGAATTTCCTTAATTTTTATCAGT<br>AAGG**TACAAAA**TTACTAATGTAGTAATTAACCAATTTTGTAAAAAAATTGAGATGTATCTT<br>TTTTTTTTCAATAAGGAAATTCCTTGAGTTTTTTCATGCGTAGTTTGCCTCCAAATACTTCCCG<br>CCAAACTCAATTTTCCCTCCAAGTAGTACCGCAACATTAGATAACAAACCTGCTAATCTCTCT<br>CCACATCTCTCTCAACGAATAAGCTCCC**TATATAA**TCCCCATTCAACCTGGAATCAGTTAGT<br>CAGTCTTCTGCTCCCCATTATCTCTCACCAGAGCACTCCTCCATCGCCTCCTCTAAGATCGCA<br>TTCTCAACTGCTCAGCCGCGCAGGGC
28	32574618	32575198	581	+	GTACGTTTGTTATTGCAAAAAGTAGATCGAGCACGTTAATTTGTTTGTTCATGGTTATCATG<br>GTAGTAGTTCGCAAGGGACAAGAACTTCTATGCACAAACGTAATCAAGGCCATAATTCGGT<br>TGGAACTGATCAAATCAATCATCGACTCCGATACAAACTGATCGTTTGAAATTATCCCATGC<br>CGCCAAATCGCCAATACGGCAATACCACGGTAACAAAATAAACCAATTACAATAACTCAAA<br>CCACGGGCAAGCCGACGAGTCGGATCCTTGCTCATCCAACGTAACTTTACAAATAATTAATC<br>TGTCAGGAACGTGTCTGGCAGCGTAGGCTGGCGAGGTGACTTCGTCGAACGCCTTCACGT<br>CGATGTCGAGCACCAGCGCGCCCGACGAACGCCACTCCGAGGCCATGCGGCCGCGGAGG<br>CGCTCCGACAGCCCCACGCCCTGGCCCCGCGCCACCACCTCCACGTCCCTCTCCCGCCGCCC<br>CGCCGCGCAGTCGCGCGGCTCCGCGTGCGCCCACCCGAGAGCCACGCCCGAGTACAGCAC<br>GGTCACGGCCGCGCGGTCGACGCACGCGCCGT
29	32944583	32945178	596	+	CGTGTTCGGCGAACAAATCAAATTTTCTGCGAAACGAACAACC**ATTATA**ACCGGCCGGCC<br>GTTAG**ATATAT**TATTTCTGCGATTTCATTTCATTCATTGTTTAACAATAGCAAATAAGTTGA

					TCTGCGAATTTCGCGGGGCCATCATCCAGTTGGCACTTGAACCTGCGCATGTTAGTTTTCCC<br>TTAACAAACCAGAAACAACAACTGCTGATCGTTCAGAGCCGGCCAATCACCCCAGC**TATA**C<br>CAGCTGCTGCTAATCATAGCAAACCTAACAATGCCCCAGCCTGTGAAAGGAACTCCCAGCC<br>AAGCAATCCATTCCTTATTCCAAGGCGACCCTGCCCTGCCTAAACAAAGCACACATGCAACG<br>CAAAAACAGCCTAAAAGCCTCCCCGATCGCAGC**TATATAA**ACACGACACCTCTCGCTCTTC<br>TTTTCATCCCAAATTAAGCACCCCCCCCCCCCCCAAGCCCTCGCTAGCTTTCTCGTTAATTCT<br>CCGTTGCCAGCATCTCCAGTCGATTCATAGCTAGCGATCGAAATGGCGGCCACCCTCAAGC<br>TCAGGATCCTCGCCGCGGCTGCCACCGCCGCCGTCGTGGCGT
30	33236531	33237113	583	-	AACTGGCAACAAAATCAGAGGATTTCTGAACTATGGACAAGATGGTAGTATTACAATCCCA<br>AAGAGTTGGGATGTGAGATT**TATA**AGGGAAATTTGCTTGCTAGTCAAAAATAAACAATTTT<br>GCTTACCAGTCAAAACAAGAAGGAATTTCACTTTGCAAGTGCATG**TATA**CTGAGATACGG<br>ATGAACAGATCAAAATATCAGATCATATGCCTGAGATTTTTGCTGCGGAGACGAGGAGTAC<br>GACGATGATGCTGGTGATGACCAGGCGGCTGGGCGACCCGACGAGCGGCGCCATCGCGG<br>GCAGCCGCCGGTCCTGTCCTCCTGGGCTGCCGCCACCGCCGAGCACGACCACCACGGGCT<br>GCTTCTCGACGGCTGGGCACTCGAACGGGCGCCGTCTTGCGAGTCATGGAGTGGATGTCG<br>TATTGGGGAGCGGATGTGGGGGCGCAGGGCTGGCGGACGGACGGTGCCCCACCTCGACC<br>TCGCCGGCCGCAAAACCATCGGCTGAAGCCATGGCAGCGACGGGCGCTCCCACGCCTCCC<br>TTCTCCTCGCCCTTCTCTTCGATGTCGCGGGCCGCGCCGTCC
31	33285904	33286481	578	-	TTACAATTTTACCATCTTCCTGTTTAGTTCTTTGCGTGTAACAAACATACTAAATGGGTTCAA<br>TGTTTTTTTAAAAAAATTATGAGGTATTGAGGTCAATTGACAGGGGCT**TATA**ACCCG**TATA**<br>CTTCAAAATACGGAGTACAAATGGGATTAGGATGATTGCCATATTTGGTCCATTGGTTGTC<br>ATGGCGATGTGCATCGGTACGTGTATGGACACTTTGAGTTGGTGGTGTGGTTTATTCTCAG<br>TTCATTAGAGAAATGTTAAGAAGTATGGGCAGGCTTCTAGGAGATGCCATGGCACAGACG<br>ATGAGACGAACGAGGTGATAAATCTGTCGTCAAAAAGGCTACCAACGAAAAGCAGCTAGC<br>GATGCAGGAAGTGCTGGTGGCGTAAAAGCGTTAGCGCTTTTGGCGGTCACCTCCTGCAAC<br>CACGCCCCAAGCATCTTCGCCGGCCAGCCGGCCGGTCTGCCTGCACTTCCGGTGACTCGTG<br>TACGAGCACCCAAAGTGCGGTGTGCGCTGTACTGGAAAAACGTAAGAAACCCACCCTCATA<br>CACGGTCAAGTCTCGAGGGAAAGGCCGGAG
32	33497786	33498313	528	-	GAGGCCTCTTGATTTTTCTCTTCCCTATTTTGGATTTCTTTTTTTTGTGCGTAATGCAGATTTA<br>CTCTTGTTTTACTAGATAAATAAATAAAGCAAAAATTGGAGTAGTTTTCTGGTAGAAATTAT<br>CTTGTTGCGATCCTATGAATCAGTTCAATTAAAACCTTAGATTCATACTAGAGCCACGATGA<br>TTGAGTCTCAAGCTAAACAAAAGGCAAGGGTTCTTCGAATAAGAACCGCTTGATGATCATT<br>TATTGAATCGGTGTAATGACTCGACAAAATCGAGAGAAAAAAAGTTGTTTTTTGGGCAAAC<br>AAAATTGGAAGGAAGAAATTTTCTTCACCCCGTAGGAACTACGAGATCACCCCAAGGACGC<br>CTTCGGCGTCCAGGGGTCACGGACCGACCATAGACCCTGTTCAATAAGTGGAACACATTAG<br>CCGTCCGCTCTCCGGTTGGGCAGTAAGGGTCGGAGAAGGGCAATCACTCGTTCTTAAAACC<br>AGCATTCTCAGAAAATTCAGTTGCCGCGAGCTCCA
33	33500803	33501310	508	+	TATTCTAACTCGAAAAAGAAGAAGAGGAAAAAATTGCAATGGATTTAAAACTAGAACTTAT<br>TCTTAGGTAAATCCATTGGGAGATGCTTCTCTAGAGTGTCCCATATCTGTTTTCCATCTTGCA<br>TACGAAAACTGTCAATTCTCATCAGATCTTCTTCCGTCTTACTCAAAAGGTCCAATAGTGTAT<br>GGATATTGGCCCTTTTGAGACA**ATTATA**CGTTCTAGAAGGCAGTTCTAATTGATCAATAAA<br>AATACAACTCAATGGAATTCTTTTTTTGTTTTTCTTTAGATTAGTTAATCTTTTTTGAAAGCTT<br>AAAAGGGGGGAAGTAAACCTGTTTTTATTTTCTTGGAAACGAGTACCCTCTTCCTCCGTGTG<br>AAGAAAAGGAAGAAATAAATCAATCAAATTACGAGAAGCCTCATAAAGCGCTTCCTTAGG<br>GGTTAAACTTCCATTGGTCCATATTTCTAGAAAAAGTATCTCATATTTTGCATTTCCATTCCC<br>ACAAGCAAAAA
34	33549561	33550124	564	-	TAATAAGAAGCTAAAAAAATGAAGCCCATGTCTTTGAAATCCAACGATCGTAATGCC**TATA**<br>**AA**AATGGAAAATGAAAGAGCCAAAGTAATGAGAAAATGACTTGTCACTGTGAAGCTAAAG<br>GGTATCATACCCTGGGGATTACGAAATAACGAAAAAGTAAAAGTGACCGAGATGCGAGGG<br>AAAAACTTTTGTTTAACATTTCCACCTATTTGTTCGTTTACCAGGTTCAGCACGAAATCATAA<br>ATAAGCTCTACCAAGGATTGCCATGCATTTGGCACTGACTTTCCCCCGCCCTTTTTCGTAACA<br>ACTCCAAATAAAGATGGCACCAAAACGACAGTTACCGCCATAGACAAGGATTCATTTGTGA<br>ATGATAAATAAAAGTTACCCATATCAAGACCAAATAATGGATCAATGAAAAATTGATCCAA<br>GGGGCTTGAAATTACTGGTCCGCCGCCTCCAGGGATAAC**TATA**TCGCCGTTCCTCCATTCA<br>GCCCCATCTAACAACTCTATTCCGTTCTGCCAGTTATTGAAACGGATACGCTGCGCCCTCTC<br>AAATGGTTCAAA
35	33558255	33558845	591	-	CCATCAGTTGATT**TATA**ACCCCCTCGCCAATGAAATTAATTCACTTTTATGAAGCGAGGAG<br>AAAGAAAGGATCAATCGAGGATCCAATATTTTATTCCCCGGAGGAAGTCTCTTGTAAATCA<br>TTGATTTAATTTAGAGTAATAAACTAATAAAACCTTAATTAAAACTACTCAACTAGCCTAAA

					AATAAAAAACAGCCTTCAATGGAAGAGTATCCGTTTTTTTGCAAAATTTCTGTAAGTTCTA<br>AATTGAACTTAATTGAACAAGTCAAGCAATTCTATTTGGTCACAAAAAATTTGTCCCCCGCC<br>ATATTTTCTTTCCCTCTTTTTGTCCACTACATCGCCTGAACCTAAGCAAAAGAGGTTCAAGAG<br>ACAGACCCAAATAAAGAAGAAATAGTAATCTTTGCCGAATAGGAAGAAAAAAGATTCATA<br>TTTCGATACAATACAAGAAGAATCGACACAAGAAAAAGGCAAAAAAGCCTTTTTCTTGTGC<br>CCAATAGAGGATTAAGAAGACCAGCAATCCCTCCTAAAAGGATTTGTTCCTTTTATTGTTCT<br>CGCCCTTGCCTTGCATTCTCCTCGTTTTGCATGAGAT
36	33563432	33563977	546	-	CTCCCAGACGGCGTATTTTTTTAAAACGAGGTCCTCGATAACGGGACATGAAGACTCCTTTT<br>TTTATTGAAATTTCATTTTACACAATTAATTTCATTGTATTTACATTACAGAATACATCGAAAT<br>TAAAACTGAATTAAAGTAAAGGATAAACAGAGTAAAATCTACTAAAAGTACCACAAAAAAT<br>TTG**TATATATA**TTATTTATTTTATTGTTTTGTATCTAGCAAAATTGTAGGGTAGAACGACAT<br>AATAGATCCTGGCTTCTCCATTTAATTCGGAGAAAAAGAGGTATTTTTGTTCATGGAACATT<br>GATAGAGAAAAAAGCCGACTATCGGATTTGAACCGATGACCCTCGCATTACAAATGCGATG<br>CTCTAACCTCTGAGCTAAGTGGGCTTACATAACAGAAATAGTGTAACAAATAGAAATATG**T**<br>**ATA**G**TATA**GGAAATCCGTAAAATGTCAGATCTTAATTATTAATCTTAGCTATTAACTAGTTC<br>GAAATTGGAAGTTCTACTTAGAAAAAAATACTAGAACTTCATAAAATAAAG
37	33571754	33572345	592	-	TGCATTTTCGCGATTTGTACCCAAAGACTAATTGAAATTGCATCCAAAAAACAAATAGGATT<br>TGGATTATGAGTACAGAGTCGCGAAGCATAATTTTGCATTGGATTAAGGATTCCAATTTTTA<br>AAATATGAGTAAAGGATCTATGGATAAAGA**TACAAAA**AAATTTATTTCCAATCGTAACTA<br>AATCTTCTTTTGTTAAAAAAGGAAATGGAAGCCAAATAGCTAAAAAACGGTAGTTTTGGTT<br>TACTAGAACCATCAGCATATTGTTTCAGCTCGGTGGAAACCCAATTCTTTTCTCAGGATCTCT<br>TAAATGAAATTAGGGAACGAAGTAATTAGATTAGATAGATTTAGGCAAATTTCTATCTCCTA<br>CTCTTTAGGGATCATCTAGAAAGCAGAGAGCCTTGGTTCCATTCAGACAAAAAAGCTGACT<br>TAGATGTTAAGTGGTGAGAATACCCATAAAGGAGCCGAATGAAATCAAAATTTCATGTTCG<br>GTTTTGAATTAGAGACGTTAATAATAATCAACCAACGTCGAC**TATA**ACCCCTAGCCTTCCAA<br>GCTAACGATGCGGGTTCGATTCCCGCTACCCGCTCCAT
38	33573885	33574443	559	-	CGATTTGATCTATTCAAAAAATATCTTAAGATTCTTATG**ATATAA**TTAAG**TATA**TCT**ATTA**<br>**TA**ACGAATATTGACTATTGTATTTTTGAGTTTGCAAAAAAAGATTTTT**TATA**G**TATA**GAAT<br>ACTTTTTTTTAGAATACTTTAGTATTCTAAAAAAAAAAGAATCGAATGG**ATTATATAA**ATA<br>ATAAGAAAATAGCAAAAAAGGAGAAGCAATTAAAAAGATTTGATTTTTCTTTTCCGATCCTC<br>GCGGCCTTTCTATCCTTCTAATCGAACTTGAAATTGGATTCAAAGTACGACCTAGTCGAATG<br>CACCTATTTCGAATCCAAAATCTTTTTCGTCTACTTCCTTCCACATGCAATACTTATTCAACCA<br>CTCTCGGACCCCACAAATCCGCTGTAAGGATTGATACATAGGACTTTTAGTTGATTGATATT<br>GCCGTAAAGTTGAAACTTTTTTAGGCTTTTTTATTAAACTGTAATTTCCTCTCTGCATACGTG<br>CTCTTCTAGAAGCA**TATA**CAATAATGCGCGGTAAAGGAGGGAAAATAGCATACTCAATCA<br>AGG
39	33586032	33586623	592	-	GATAGGCGAGAGGTGTAAGCACCGCGAGGTGTGAAGCGATCTCGTACTAAACGAATGGA<br>ACTTTCCAACCTTTGAGAAAAGGTCCCATCCTTCAATATCATGATTGGGTCAACCAGGCCAG<br>ATCATAAGTGAAATAGTTTGATCTGGTCGACCAGGTCAGGCCCGATCAAGCGAATTTGTAA<br>AGTTGTAAGATGAATCACCTTTTTGTACTATTTATTTCACGGTTTTTCGGGAAAATCTTTTTT<br>CATTTTCCCAGCGTCCTAGAATTGCCTTTCTGTTTTTTCCTTCTCCTTTTGATTTTCGTATCATT<br>ATTATTACTTTGGTTTCTTAATAGGGGGATGATTCATCGTGGAACCCGTTCCCCCAACGAAT<br>TGGATTTTCATTTTTTTTTATTTTTGTTTACC**TATA**AATTTTGTTTAGAGACTGTTTTTTTAGA<br>TGACGGAGAGAATGTTTTACCAAATCCAGCAGGGGATCAGGCTCAACCCGCGGATGCACC<br>CCTTGCTCCTGCAGTGCAAGTCCCTGCCATTTTGAACCCACCCCGGGTTCCATCGATCCCGT<br>CATCCCTGGATCCAGGGAGAACGCCAGAAGATCG
40	33727945	33728534	590	+	AATAAATCTAAACATAAATAAT**ATTATA**TTTTTATTATTCTAGATTGAATTTTTATGTGACA<br>GATAGTGTAATCATACAGATTTTTCAGAGTAATTCGTCTGCAAAGACACCGAAAGGAGACA<br>GATTCTCGTTAGAAAGAAAGGAAAAGAACACGTACGCGAGAAAGCACAGATTCTAAACA**T**<br>**ATA**GACATAAATCTTTGAGGGACTGGTGTGGTGCATATTGTTCAAAGATATTTCCGATCGG<br>TGTG**TATA**AATACATAGCCCGCGGCGACGAGCAGGTTACGTGCCGATCGAGTTGGAGGG<br>CAAGAAATCACAAGAAAAACTGCCTGTGCGCCAAGGAGACCAACACAAGACAGAAAGAA<br>GATGATGAGGAGGTCGATGTTCCTTCCCGTCTGCGCGCTCGTCCTCGTCATGCTCTGCGTCT<br>GCGTGGCGAGCCACATGGACGTGGACGAGCGCCGAGGCGGAGCTCGCGCCTACATGGGC<br>GGCCACGGCAGGCCCGTGGGCATCAGACCCAGAACCAGCGGCAGCCCGCGCGGCCTCAG<br>CGGCGGCACCTGGGCGGCGTGCGCCGGCTCATCGCTGCTCGCCGCCGC
41	33748436	33749033	598	-	GAAATTTTTGGACCATAAAATTTTGGGAGGCCCAAAATGTCATGGATGGTAACATGTGGGA<br>GGGCCCAATCTGCCATGGCTGCTTTCATGGACCCACCGGGCAACCGGACCTTGGGCCAAAT

					TTACTACGCCAAGAAAAGGGCTTAGATTAACCAGCATAATTAGCTCTGCTAATGGGACACC<br>GTAACTAGATCTTTACCCGCACATGCACCGAAAAACTTCCTCACACGGCCGAACAAGATCG<br>CCGTGCGAATTTGCACACAACGGCACGGCGGTAGGCAGGCAGCGCAGCAGGCCGCCGTG<br>TGTGCCACTGCTGCAGCCCGCCGGCATGGGCCGCCTGGCCGCCAACACGTCCAGTACGCC<br>GTTCGTTACCCAAAGCGATCGTCAAATCCGGTCGGACCACACGCACGCTAAGCACAACGCG<br>AACGCATCCGGCCTCCTCCTCTCTCGCTTTTATTATTTTTTTCTTCCTTCCTTCCTTCCCCTCTG<br>CCACTCTTCCGCCGTTCCACCCACCTCACGCTCTCCTCCCTCTCCACAAAACCACCGCGCCCT<br>GCCGCCCTCCCGGCCGGCCTCCCCAACGCCTCGCGAGGGGCACCGC
42	33755037	33755589	553	-	TTGTTGTCACCGCCGGACATGCACGGGAGCCACACGGTGCCGTCGGTATGCAACCAAATCA<br>GTTGCAATTGGGGATATGGGGACGAATCTATTGATGATTTGGGCAGAGGGAGAAGGAAA<br>TGCAGAACATG**TATA**ATGGGAGAGGAGATGACGAACCACGTTGGGTCAGCGAGAGAGA<br>GACAAGGAGCGCCGGTCACCAAGGACATCGGGCTAGCAGCAAGCGTCGGCGTGGTGGGT<br>GTGTTTTTTCATGACTAGTATTGGGGAGATGAAGACACAGAAGAAAAACTATGAAACGTAT<br>CCCGTCAAGGAACGGGAAATTAATAACCCAACCGACAACTAGATCAAACCAGCAGATGTG<br>AAACTCTGTCCTCCAAACCATATCCTCGAGACATTATGACACTCATCTTTGTCTCTGCTCGCA<br>TCCCCTTCCTCTTCTCTCCGTTTCCTCCTCCCTCCCGTCCTCAATGACTCCGCCCCACAGCCGC<br>CGTCCTCCCCCCTCCCTTCCTCGACTCCTCTACCCCACAGTCGTCATCCTCCCCCTCCTTCCCT<br>TCCT
43	33765168	33765751	584	+	TAGAAACTAAAGCTCCCGGTGAGTACCAAACGAAGTCAGTGTAGTTAGGCTTCTCATGACT<br>TTTCTTTCTCGTACTCGTACAACG**TATA**ATGAAATCTTTTGCTACAAATAATTGACTCAAATT<br>TGTGCAGTAGTAAGATTTTAGACCTTCGGTCATGCCTGTTTTCGAAATAGTTGCATCAGTTG<br>TCATGATGCATCATTTGGTGACGACTACTGGCGCCAAAAGAAAAGCCAACACTCCTGGTCT<br>ATTCACAGAAAAGCATCACGTAAGCTTTTTTTCTTATCACCACGAAAACACAGGAAGCATCA<br>GTGGCTTCCGAAAACCAAATATTCCAAATCCGATGCGAGCGGCGAGCGGGTCCGTCTACG<br>AGCGGCGTATCTTTGCTTGCGGCACCAAAATTTCCCCGCACGACAAGGTTCCTCCCCCCAAC<br>ACGCGCCGCTCAGACTCGGCTAGCTAAAGTTGACGATCCGTTGGCGCGGGAGGCGTGCGC<br>GGAACACGACGACAGCGTGGAGGGTGGTGGCCGCCGCGGCCCACGTGTCTTTACGCGCA<br>GGCCGCCTGCTGCCGATACCGATGCCGACGCCGA
44	34384446	34385041	596	-	GCCAAGAAAAAGACAACGAACATAGGAGATAAAGAAACTGGATCCCAGAGGTGAACGCG<br>AGGGTTCTTGATGAAATTCCGAAGCAAGAGATAAGCGATCGATAACCTTTAGCAGCGCCAC<br>GAACAGGCGACGAGGCGCGAGCGAGACGTTCGTCGAAATGCCCCAAAGAATCCTAACCTG<br>CCAACAAAAAAGCGAGAACGAACAAAGGAGTTCAAGAAACTGGATGGGAGAGGCGAACG<br>CGAGGATTTTCCATGAAATTCCGCAGCAAGAGATAAGCGATCAATAACCTTTAGCAGCGCC<br>ACAAACCGGGCGACGAGGCGCGCTCGAGACGTTCGTCGTAATGCCGCAGTGCCGCACCAA<br>TGAATCCTCCTAACCTGCCAACAAAAAAGCGAGAACGAACGCCGACGAGTTCAAGAAACT<br>GGATGGGAGAGGCGAACTCAAGGCATTCGATGAAATCCCGAAGCAAGAGATAAAGAGCG<br>ATCGATTACCTCTAACAGCGCCACGAACGGGGGGCGACGAGGCGCGCCGCGCCCGCGCTC<br>CGGTCGCCGCGCGCAGCCGCGAAGGTTGAAGGGGAAAGCACCGCCACCGCCGCAGCC
45	34400801	34401392	592	-	CGAATCGGCTGTCTGATTTTTTTGAAAAAATAGGACGTTGTTTCCTCTCT**TACATAAA**AAA<br>AATATTCCAATAGTTAATAAAACAATGTTTCTTGAAAACAGTGAAACACAATTTAATCACCA<br>GATGATACAAATGATTTAAAATTATTAATTAAAACATC**TATA**GATCCGAGTAAAAAATGAA<br>GACAAAAAATAAAACATCATCCAAAACCTACTGCAACATGTTTTTCGAACTAATGAAACATC<br>TAGATGCATGAAATGAAACATTGAAAAATTTACAACGCATCCATACACTCTTGTGGTGCTCC<br>TCCCTTGTGCACTCCCGACGGTGAAGCGACGACATCGTCCTCGACCACATTACCCTTCTAGA<br>ACGTGAGAGAAGAAGAAGGGCCACCGTCATCTAGATCTGAGCCTTCTAGAACTCTCGTGG<br>ACGAA**TATA**GTCTCCTCGCTATCATACTCCGTCGTCGCCCTGAGCCTGGGTCGTCTCCACCG<br>CGGTCCGCAGGGACTCCGGCGACAGTAGAGGAGGAGACGACAGCAGCGGAGAGGATGG<br>CGGGGCGTAGGGACAGGAAGAGGAAGGTGAAGGGCAGCAGAG
46	34491677	34492245	569	-	GTAGGTTTGCTGTGTTCAATTTGTACATTTTTTTCTTTGTTAAC**ATATATA**TG**TATA**TCACA<br>C**TATA**TTAACATATCACTATTTTTAGTATTAACATAACTATGATATTTTAGTTTTGTTTTTTTC<br>TTAAACTGTGCATCTTTATCTGTTCTTTGTTA**ATATATA**TCACAC**TATA**TTAACATATCACA<br>TTATTATTTTTGTGTTAATTAACCTGC**TATATAT**CACACTATTTTTCGAGTTTGCTGCTTAAT<br>TTTATGCACCTTTCTCTATTCTTGGTTAGCATATTTCACACTGTGTTAACACATCACACTATTT<br>CTGTTTATTAACATATCACATTATTTTTTAGTGTTAACCTATCACACTATTTTTTTTAAGTTTG<br>CCGCTTAATTTATGCATCTTTCCATGTTCTTGGTTAAC**ATATAT**CACACTTTATTAACACATC<br>ACACTTTTTTTTTTGTTCTAGCGAGTAAGCTGGGTCCCAGCCGCGAAGGAAACTGGTACAT<br>GTTTTCACCTAGAAACCGGAAATACAACAAAGGCAAAAGGCCTAGCCGAAGCACCGGTCA<br>ATTGGG

47	34494003	34494581	579	-	**TATA**ACAATAGAG**TATA**TGTTTTTAATATGATAAATAATTGAAC**ATATATATAA**AAAATC<br>AACGGTCTCATCTATTAAAGCC**TATA**ACATCTTGCACCTGGGCTCTAGT**TATA**AGGGTTTG<br>GTGTTGTGAAATTTGAACTTG**TATA**TTTGCGTAACGATACATTTGTATTAATTTACATTGTT<br>ACTTTTT**TATAATTATATAT**TCTCTCGAGAAATGAAAATGCTTTCCCTAAATAACAATGCA<br>TTGGTTGCTGATGTTACTAGCCAACAAAAGAAGGTGACCTCCCAAAGTGATGAGCTAGC**TA**<br>**CAAAA**TTTTCGGTGCACAACGCAAGCATGCCTGC**ATATATA**TCCCATCCACACACACACA<br>CCCTTATTGCGTGCGTTCGTGCTGCCGCCTACGTGCATATGCGTGCTGCTTTCGCTGTACGT<br>GATCGAATTGGGAACTCTTCTTCTCTTCGATTGTTGCATGCACATACCGTGCATGCCCCATT<br>GCACCAATAATTTCCTCCGATGGCGACCTCGTCTCATCAGCAAGAGGCGAGATCGATCGTC<br>GTCGTCATTGGGCGGCGGCGGCTGATCGT
48	34546000	34546575	576	-	TAATAACGAAACTAATCAGATAATTAACATTGCTAATTCTAAGTGGAGACTAATTCACGACA<br>CGATTCACTGACCTGCATTGTCCTGGATCTTCCTGTAGCATCTCGCTGAAGCGAGGAGCGCT<br>ACAGCTGCGACCAATTCTGCAGGTGGCGATCTTCTCTTGAGCCTCTGGATGGCTTCGTTGCC<br>GAATGCTGCCGCTGCTTTTCTCCCTGC**ATATATAA**ATCAAATCAGATCAACGGAAGATCG<br>GCAAGTAATCATCGATGATGTTACAAATCAATTAATGTTCGATCCAATCGATCGACGTTGAA<br>GCAGTAACATACAGTTCTTGGCGAGGACGTAGGAGAGACTGGCCATCCTGAAGTCCTCGG<br>CGATGGCGAAGGCCGTGTCGTGCTTGAACGACGTGAACCACCAGCTTGAGGCTCGCCGGT<br>GGCCCCTCGCCATGAGGCGGTCGCCGCGCGCCTCGAGCTCGTCCGGCTCTAGCCCCTCCTC<br>CTCGAACATCGCCGGCTCGTACGCCGGCGCCGCCTCCTCCTCCTCGCCGCCGCGCCGC<br>GTGATCGGTTCGTAGCCGCGGCGGCG
49	34896918	34897507	590	-	CCGCTGTAGAGAGCACTTAGTACCAGGCACACCAAGGTAGTCCTTTAATTTAATTCTGATGC<br>CATTAATTAACTCCGACCACCCAACTCCTCGGACATTAAAAGGGGGAAATTTTTTTTTTA<br>GAAACGCTTTTTTCCCCTTTATTTCGAACAAGAAAATTGAGGAGAAACTGACGAGACACTC<br>ACTGTCTACGAACGAGCGCGGCACATACTACATTGCACAAATCTACGACCATCTCCTTCAAT<br>TCTGGGTCCTAAATCAGGGTGCCAGATCAAACCCAGGTAATCAGGTTCCAATCTCGCCCCC<br>AAATTCCAACCCCATTTGCATACAAGAACGCAATGGTGGAGAGAGAAAACCAAAAAAAAA<br>AAAAAAAACGGAGAGACAGAGAGTCGAGTCCCCAACCTTTCGCCAGGCAGAAGAAATCCG<br>GTAACCCCCCCTCCTCCTCCCCTCCACCACCATTCGCCTCAAGAATCAAAGATCCAGCCTTCC<br>GGCCGCCGAAATCCCGCCGCGCGGGGGAGGTCAGGTCACCGATCTGCTTCCGAACACGCC<br>GGCATTTCGTCCCCCGCGACCCCGGCGGCGGCGCGGGGA
50	34977772	34978310	539	-	TGAATTTGTATGACTAG**TAAAGATT**TCCATTAAAAATGCAGAAATTTCACAAGAAATTTCA<br>GAGAAACAGTTGAATTCCCACGGAGAGATGAACATTTTCGCACCTCCAAAAACCCCTCACC<br>ACGGACGAAGCAGTAATAAAACACATAGTTCGTTATCCTCGATCTCCTGTTTCCTGGTGGCA<br>TAAGCATGACATGGAAATTCCATCTAGGTTTTCAGCATCAACCATCGATGCTGGCTAACAAC<br>AATCTCTGGGCTAAAATTTTGGGAATCGAAGTTGCTAAAGCGGGGCAAGCTTTGGGCCAG<br>AACCGGCAAGGTCAGTGCTGGGCTAGGCTAAACAGAACTGGAACCGAGACGGAGGAGTA<br>AGAACCTGCAACAAGGAACCCCCCCCCCCCTCCTGCGGCGGCTGCATTGGCCGGTGCGCC<br>GCGCCTGAACGATCCCCCTCGTCGCCGGCGCCGCATGGATTCCCCTCCTCCAGCCGCGTCCT<br>CGCCATCGCCTTCCTCCCGACGACCCCGTCGGCGCCCCGCCGCCTCGCCGT
51	35053715	35054312	598	-	GTTTCGTACCTTGAAGATGTCAAAAT**ATTATA**CTTCTGAAGAAAAAAAAAAGGAGTCATC<br>GTCAAATTTCAACAGTAAAGCTTGTAGAGTCGGCCAAGGACAGTGAAACCGACCCCTAGA<br>GAGGGAGTAGTAAAGTCTTCC**TATA**TGAAAAAAAAAAGGAAGGAAAGGGTGTAGTATTG<br>ACTGTACTAACTTGAATGAAGCAAATAATACGATGGCTGGGAAGTCTATTTGTAACAAGTC<br>CCGGATGACTTACTGGTGGGGACAATGTGTGATTGATCGCTGCTTTTAGCACACTGTTGGT<br>CACATAAGGGCATTAGCAGTGCATCCGGCTTGTTTCGTGTGACCATGTGCTCGACATAGAC<br>AAAAAAGTGAATGAAGCCACACATAACAAATTTTAATCTCCCACAGTACGCCGGCCTCCCTC<br>TCCCCTCCCCAGGTCATTCCCCTAACCTCCCCAAGCCGACATGTCTCCTCCCCTTCCCCACCA<br>CCACTGGCACAACTCCACTTCTCCCGACCGTCGGTGCAGCTCTCCCTCCTCTCCGTCGCTCCC<br>CCTCCCTGTCGGCGCCTCTCAATCGTGGACGCCGCTTGAGCCCAGCTC
52	35092265	35092849	585	+	TCCTGTGTTGAAGCCTATGTTAGATAATAAGTTGGCATAAACCGAATTTTCATCATGGCACC<br>TAGTTCTGCACGGAAGGAAAAAAAAAAGCTCATCCAACTGTAGCTCAACAGCAAAACAAACC<br>TTTCCAGACTCATGAAACCAATTCGTTACCAGCATAAATCCAGATTATCAGTCA**ATATAT**TT<br>GTCGCTCCC**TATA**AA**ATATAA**ACCTCTGGACCACTGTTCTTTTCAATACATAGTGTGGCCA<br>GCATCAG**TATATAT**GAAAGAAAAAAAAAATCAGGCGTGTGATAAATG**TATA**GCAAAAACA<br>AATTAAAAACCGGGGCAATTTTGGTAACAGAGTCAGATAACTGATACTATGACTGCATCAC<br>AATCCTTAACAAATTCGACTCAAAACTAAGCAATTGCATAGACAATTAGGATTTTCCGAACA<br>ATTACCGTCGTTCTGGTTAGCCGTAGGCGATCAGGCGAACAACTCTTCCCCGCGGCTTGTC

					GCGCACCTGCACCGATATGTCAGAGCCGGGGATCTCCCTCAGGCCTCAGCTGCATAGGCG<br>GACGACGGCCGCCACGGCGGCGGAGAGCCGTGTCCT
53	35375036	35375528	493	-	ATACATCATTTGATTTTGACCTGGTGACAAATGAAGCAGTGATGATTTGTTTGGATTATGTG<br>ATGAGTATTTTTAGTAGTGATCTTAGTTTTTGTTCTCTGAAATATTTCAGTCTAGTAAGGGCC<br>TTGTGCTTCAA**ATTATA**GTG**TATA**AGCAGGTTGATGCTCTGATCTAGCTTTGGGAATCTAT<br>GAGAGCAGAAAGCTATGCTATGAAGTAACTGAATATTTTTGGTTATTGCCCTGTTACAGAT<br>ATGTTTATCATGTTAAATTATTTTATTGTGG**TATA**GTTTTGTTGTTTTGTAAATTTTGG**TATA**<br>TGTTATTTTAGCATTTGAAACTTGCAGCATGTGCAGAAGTTTGCATGTTTGTGTGGCTGAGT<br>GTTGA**TATA**CAGATTTGAAGGATGCTTGAGTGTGGCCTGCTTTTGTGATATTTTGTTTGACT<br>GTGTAGGTCTATCATTTAAAAAAAATCTGATTGTATTTTAGCTATTTGTAGTTTTAAGA
54	35464723	35465310	588	-	TTCTACCTCCTTTCTTTTTCATGTATTTTTCTGATCAGGAAAAATAAAAAATTATGTCAGTTAT<br>TTTGAAGTTATTCTAATATCGTACACACAAAAATTTGCAATTATTCATCTACTACTGGAATTT<br>GGATTTATTTTATCGATGCAAATTGGATTTGGATAGAAGGGTACATTCTTTTATTTTAGATA<br>GAAGAAAAGTTTCTTCTATCTAAAATAAAAGAATTTTGCTGATTTATTTATTGC**TATA**TCCA<br>ATTTATGAAATTGATACACCGTTTAATTGATATCATTTAGCAAAATGAAAGTTATGTAAGGT<br>ATCAAAGTTTGACTGCAAGCACTATTTGAGAACGTAGATGTAGTATTTTTTTAATTCGTACC<br>TGGTGCTTTAATTTGAACTCAATTAATTAC**TATA**CAGGCTTAATAACAACACTTTTCTACTG<br>ACACGGTCATGCATGCCATAATCAGATGTTTCAGAAATGTCTGCTAGCCGACAAATTAAGA<br>AGC**ATATATA**CTCCAGGTCGTGTTAATTTGTAGACACTTTAATTCTCAGAACAAATTGCGC<br>TGCTGAAACATGCGCGGCCGCGATAATCC
55	35723764	35724358	595	-	CGCTGCTCCTTTTTCTTGGTTCCTGCCTCTCCCTCTCTCCGCTCGGTTTGCTCCCCTCCTCCAC<br>TTCTTGTCGGCCTCTCTGCTCCTTTCGGCCTCGGATTCTCTCTGTGTTTTTCTACCCCCATTGC<br>ATCCGGTTTAAGCTGAAATCTTGAGCCATTTTGGGATTCATTCAGAAGGAATCCACAGTTTG<br>GATTGGTTCACTACTCGATTTGGTTGAAGTTTCTTTCTTTTGCGGTCGTTTTAGCATGAGGA<br>GGGGAACTGAAATCTCCTGCTTATTGTGACAATGCTATCTCTGAACAAAATGCATTTTCTCA<br>AGT**TATA**TTCCAGCAGTACGAGCGGGAGGTAACGCGCTTCAGTTGTGAACCCTGCATGCC<br>TTGTGATTCTTGCGGTTTTTTTTTTCTGAATCTTACTTGATGTGTTCTTTTCTGGCATGAAGG<br>AATGGCTCCTTGGAACACCCTACCCGGACGTCTTCCCAGGGAGCTACCAAGACGAGCCGG<br>ACGGCGAGGCCGGCCGGGCCGGACTCCGCCGCCGACAGGCCGTCGACGAAGTCGCCGCC<br>CGCCGGGAGGAGCCCCAAGGTGGAGCGCCGCATGACGAT
56	35877995	35878586	592	-	CCTGCACCTTTTGCAGGTGATAGTGCTGATTAGAAAGTGGTCTCAATTAGGTTAGGTGTTG<br>ATCGAGTGCCTAACACCCTACTAAATTCCACATATTGGTT**TATA**TCAAAATATCGTCGTAGA<br>AAAGTCTGCAACTCTGAACAGTTAATCCCCTGAATTGCAGGTCTAAAGGTGAATGTTTTGC<br>AGGCACTGTTACTTGGTTAGTGTTGGTTGGAACTTGAAAAAGATTCAAGTTTGCCCCCCTAC<br>AGATTCATAGGCACTCGGAAAT**ATTATA**GAAACGGTGAACAACCCATGCAAAACTTCATA<br>ACCACCTTTCTTACGGACATGGTGACATGATTGTACCTGAAAATGCATGGCGCCTGAGGTG<br>ACAG**TATA**AACCCTAAACGATTAAATTATGGTAGAGTTAGTTTTTTTTTCCTGTACAGTAAT<br>CTAACACGCTGCATAGTGTTTTAACTGTATGTCTGTTTTTTTAAACCCATGCGAGGGCAGCG<br>AGGCCAGAGCTGCCGGACATTTCTTGGTGGGAGTCCAATATTTTTTGCCCTGTAAGAAGAG<br>AGGAGGGCCCTCTGAAAGTTCTCAAAGCTCGAGGTGTTCT
57	36056999	36057577	579	-	GAGAGTGAGTGAGCGTAGAGTGGAGTGGAGTGGAGTGGAAGTTGATATCTTCATGAGTG<br>GACGAACCCGTCGTGCTTTTTGAAACTTATCTGCATTGTTGCATCGATCATTGCATGGTTTA<br>ATTGGTTAAGGGATTGGAAGGTGTACTGCTTCATCAACTTGAAGTGTTTCATTGGAATGGA<br>ATGATGCATGTTTTGAGGCGGCTGTGCTCTCACAATTTGTTCGTGCTCTCAAGTGATTTTTG<br>TTTTAATCATAGGTGATCTTTTTTTTTCTGATTTGAGTTTGACAGGTAGTCGACTTCGACACT<br>GAGTGTTGCAGATATTTTACGAATGTCCTTAGTCAGAGTAAATGTTGAAGATTTTTTTTTCT<br>GTGGGGCTCGAGTTTGGACCAAAAAACTGATCCACTCAAACAATGGCCCAGTAACAAGTAC<br>AATGGGCAGTGATGGAGAAGCAACAGGGCTCAAGGCCCGCACACCTTCACGCTACGCGAC<br>CCAATTCGACGGCCCCAGCCTCCCAAGTCCCAGCACCAGGGCCAAATCCCCAACAGAAATC<br>AAACTCGGCCGGCGACGGCGAGTTCCCT
58	36088816	36089410	595	+	TTCAATGGCGCACACGATTCAATGCCACTTAAATCACCGCGTGTGGTAAGAAGAGGGGAA<br>AAAACGCAAATTGATCGGTTAACCTTCGCAAGCTGTTTTCGTCTCCTCGTTGGGATGACGCG<br>TTGCTCTTCCATCCATGTACGCTCGAATCAACAAAAACTGATAAAGAACTTGTCTATTTTTTT<br>ATCGGCAAATGTTTTACTCGGCAAAGACAAACGTAGCAGAGATCAATGTGAAGAATCATG<br>AAAGATTCGACAATGACCCGGGTTCTCTGGAAAATGAGAAGAATCGAAATGCAACGCGAA<br>ACCGATCAAACTCGAGCATCTCTAGCCATGGTTCCCCATGAGAATATGAGATCATAAGCTA<br>CAACGAGCTGCTGCTTGCTCTCCTCTCCATCTCGAGGAATTGCCCGAGCGACGGCGCGGCG<br>CGGCCGTTCTCCAGGTGGCTCCGGAGGCAGAGCGACAGCAGCAGCGCCCTGACCTTGCCG

					CTCTTGCTCGTCGCCCTCGCGCTCCGGCGCCGCCGGCTACGGCTCTCCATGGCGTCCACCAG<br>GCCGATCAGGCTCGCCAGCGTCATCCCGCCGCCGGCGCCGCCGCCT
59	36110385	36110848	464	+	AAAAAACGACCAAATATTTTCATCGCAA**ATATAA**ATTTCTAGTGCAACAGACCCAGGCTGC<br>GGATGAGCAGGTGGGAGACACTCACCGCGTCAGTGGCGTTGTCGGCGTCCATGTCGGCGC<br>GCAGGCGGGCGTAGAGCTGCGGGCTGCGGTACAGCGAACCAGGCGCCGGGCGCGAGAT<br>GATGCGGGGCACGCTGTCGAGAGAGATGGAGCCCATGTAGAGCAGCATCGCCGCGACGT<br>ACAGCAGCGGAGCGAAGAGGAAGACCGCCTGCCGCCGCAGCAGCGCGGCGAGCACCACC<br>CTCGCCGCCCTCCGCACGGCGCCTCCGCCCCGCACAGCTCCGGCCGCGCCTGCCCCCGCCC<br>CCGCCCTCGCCGACCCGCCCTTCCCCGACGACCTCCTCCCCGCCCCCCGCCGCGGCGAAGA<br>CGGCGGCGACGGCACGGCGCCGCTCCCGCCGCCGCCGCCGCC
60	36376917	36377475	559	-	AGATTCACCGATTGATAACACCATGTTTTATCTGAAAGGCATATGCACTAATGTGCGATT**TA**<br>**TA**TGTCTGGAA**TACATAAA**TTTCATGATATTTTTAATAGAAATCCACACGATACTGTAGTT<br>CGTTATGCGCACAACATTTTTCACCCATGCCAATCAGACTAGGGTGGGTTGTCTCGTGGGC<br>GGTTTAACTTCGGCGTCAACAATATCTAGTGTAGAGTTTTGCGTCCATTTCTGAAATATGTT<br>TTTCGAAAGGGTTAGAATGTAAAAGATAGCTGCTTATGTAAAACTAATCCTTAAAATAATAT<br>TCTAAATATCGAAACCATTTTTTGGCTCCACACATGACCCGATCGAATGGTAAACACACAGG<br>CTCGCGGTGAGAACGTGTGACCAGCGTGCAGCCGTGCACCGGCTTTGGTGCGCGCTCGTTT<br>ATTATTCGGCCCTGAACGCTCCGGTGACGAACCTCGTCTACGCATCATGAGTTTCTTATCTC<br>CCTCCGTGGCTTGACCGCTCGAGGGGCCACGAGACTCACGAGCGGCGGCCGCCAGCCAGC<br>CGATGC
61	36427148	36427603	456	-	TTTCTCGAGTTAAAATTTAGCCTATGAGTTTTTTGGCATTGACTTTTTTTTTACTGAGTTAAA<br>ATTTATCCTATGAGTTTTTGCAAAATTTCTCTTCTGGCATTGACAATTTTTTTTTTGCTGAGTT<br>AAAATTTATCCTATGAGTTTTTGCAAAAATTCTCTTCTTTGTATTTGCAAAATTTCTCTTATTT<br>TTTGCATTGACATTTTTTTCACTGAGTTAAAACTTCTCCCATGAGTTTTTGCACGCTCAGACA<br>TTGCAGTTTTTTACATTGACAATTTTTTTTTGCTGAGTTGAAATTTTGCTATGAGTTTTTGAAC<br>TCTTTCAAATATGGTCAATCCTTTCAAGAGCTGAAAATTCAATGGAACATTTGACGGCGAGC<br>ATGGGCAGCTGCCCGTGGTAGCCGGATGGTGGCTTTGCCGTTGCTTTGTAGGACTAATTTT<br>AAATTTTAGAATTGC
62	36503588	36504163	576	+	AAATTAGACTTGGTGCAGGAGTGGCGTTTTAGACTTAATTCGCACTTGACAGCTACTAGGG<br>GTTTTACTTTTTCTTAGAAGGGTCTGAAATTGATTTTTCTTAACTAAA**TATA**GTTGAATTGTA<br>CGTACTTCTAAGCCTGGAAGCAACTAAGATGCAATCGGTGAACTTCAAGTCCAGCATTTTTC<br>TCAAGCAAAGCACCTTAAGGACAAATCCTTTCTTGCATCTGTGACTGTGAGTTCGCCATCAG<br>ACATTCAGACTGCTAATTCTCCCAGTACTTGGCACTGGCAGATCTGCCGATGTTTCCAGCGG<br>AGCAAGAGGCTGGATAACTGCTACTAGCACCACTCACGCAAGCGAAAGAAGCACATGAAC<br>GCAAAGCACCAACGTATGTGCACTGTGCAGCGCAAATCGCCGAGATGCCGTTCTGTAAAGC<br>GGCGAAAGATTTCGCTTTTGCTGTGCCAAAAAGGCTGCTAGTAGAACATCCGATGCCATCC<br>GGACCGGCCTACGCGTGACCGCCACGTCGGCCGACCAACGGATCGCAAGCGACCGAAAGC<br>GCGCTGCGCCGCCGCTGCCGCCAC
63	36798702	36799236	535	-	GCAGCACACTTGCATTATCGATTACAAAGTCTTTGACCAAATAATACTTATTACGTTTTAATT<br>TTAAAATTATTTAACCAATAAAATAATAAAGTGAAATTCCAATTTGAGACAGTATTACAGTT<br>TTTATT**TATATAT**TTTAAATTATTAACTTAATAAAACATAAATTA**ATATAA**AATGTATTAGA<br>ACATGTCTAC**ATATAA**TTTTGTAATGATTAATTTTGTTTTTAACTATTAATTAATAAAATCAA<br>ATAATTTTGGGAGTACACGATAAAGGTGGTAATGAATTTGTGTGTTGACCGCATATGGCAC<br>CTCCCAAGTGGCACAAATATT**TATA**TTTTTCACTAGAAATTTAATGAAGACATGGGTTTGCA<br>GCTAACCAGGGGATGCATTTATCGATGAATGCTTATGATGTGTGTTGTGATATGTGCATGT<br>GTGTCATTGCAAGTTGGGAAAAAAAAATGAAACAGTTTTATCCTTGATACT**TATA**AATGAAG<br>TGTTTTTTTGGTGCGGAATAAAAAAATGAAGTTGATCACGC
64	36892174	36892762	589	+	CTGAGGAAGGAAAGTTACTTGTACAGAAACAATCAAAATCAACTTGCTGCAAGGGTGTGC<br>ACATCTACTTTCTGTTTCTTTCTCTCCCTTTCATTCCATTACAAGTAGCACTTCGATCCTTAATT<br>CGTCTCTGCCTCTCTCCAGAAAAAAGAAGAGTAATATGA**ATATAT**GCATTCTGGTGTCATT<br>AATAGGCACCATGTTCCCGACATACCACTGTAAATATGAAAAAAAAAACTGAAGAG**TATA**<br>ATTGGGAGGAGGGGAGAAGAAAAACAACCACCAGGCGTCTCCGTCATCAACAAGGAGAG<br>GAAATCCAAGT**TATA**CTCGTACAGTGATGGCGATGCAACTTGCACACCCTCGCTTGGCGGC<br>TAGCCGTGGCTCACGTTTCCGCTGTCGCTCGCGCGACGCCGAGGCGAGGCGGGGCCGGGC<br>CGCGGGGGTGGCCCAGGCGCGACCTAGGACTGGTCCTCGCGGCCGCTCGCCACAACGCCG<br>CCCCCGTCTCGACCGTCCAATTCGGGGGCAAACCACCAACCGATTCCACCGGGGCGATCGA<br>AATCTCCTCCGGCCCGTTTGGCCGGGTCGATCCCAACACCGG

65	36962635	36963223	589	+	GAAGATGATTTTTTTAAAAAAAAAATTCAAA**ATATAA**GACAAG**TATA**TTTTTTAAATTATTA<br>ATTACTTGATTAAGGCTATTAGTTTTAGTCTTCACTATTAAAAAAAATATTGTACCAGATGTG<br>TTGGAATTAATTGGGATTTACTAAGGGTGAAGGTTTTATGAAAAAGATAAGCCATAAAACC<br>TATGATTATGTC**ATATAATATA**AGTCATAACAATCGGTGTCTTTAACTTAGGTCATAAA**AT**<br>**ATAT**TACCTCCGTTCTAAATTTTAG**ATATATTATA**AGA**TATA**CTTACTCTGTTCTAAAATA<br>AATTAACCTCGTACATGATGCGA**TATA**CGAGTT**TATA**TCTCGTATGAGATTAATTTATTTTG<br>GGACCGACGGACTAGATATTAGTGTGAAATTTGCACAGACGAAAAGGAAATACTAGTAGC<br>CAATAGAAGAAGCCACAGGTGCGCTAGTTTAAGTTGGGCGTGGATGGGGAAAGCAATAG<br>ATGCGGCCAGTCCATTTCATCAGGACGCAGTGTCGCAAGTTCCAAAGAGGCCCACCACCGG<br>ATTGGATTGGCCGAGACCATCATCACCGCCATGATCACCA
66	37150985	37151580	596	-	GGATAGAAAGTAAGGAAAGGGAAGATGGGAGAAAACAAAATAATGAGAGAAAACTAGG<br>GGTTTGAATAAAAGTAGATTTGTGTCTTGATTTTACACCCTATTTCTTAGCACTTACATATCA<br>TTTTTAGG**TATA**TGTTAGATTTTAGTTTTCATTTTTTAAAATATTCATCTGATCGAAACTATG<br>TATTGCTAATACTCGATGTTACACAGGCTAAATACCCAGCGTGCCAACGTGCGCGCTGATTT<br>TCTAGTCTATTCTATCAGCAGGCCAGCAATGTTTGGTATCTAATGGGCCCGTCGGACCACTT<br>ATCGGAGGACTTGACATTGGGCCATATCACTTACTGGGCAAGATTGGGCCGATCAGCGTAT<br>TGTCCATGAAGGCTGGAGAGAACGACGAAACGGGGCTCCAAAAGCCCGCATGTGCTCATA<br>GGTCGTTATGGCCTTATGGGCCTTGCCGGAATTAGCCAATTAGGCCTTACAATTCCAACGTC<br>CGACGTCGTCTATTAGAGACGTTCAGCTCATCTCATCATCCCCCAAAGGCCTAAGCCCCCAC<br>GATCCCCATGCCGCCGTGCAGCGCACTCG**TATA**TTCCACGCGA
67	37419706	37420298	593	-	TGATCCATCCAGGAAATTCATTTCTGGACTTATGTGAATGATGTAGATCT**TATA**TCTCATGG<br>ACTTTCAAATTTTATTTGAAGAGGTTTACTACTCTCTCCTAAGTTCGAATATTTTGCTTCCAAC<br>TAAATCCATATGCACT**TATA**ATAATTCAATTAGAAGTACTGTTTGAAAACCTTGATGAAATT<br>AGTAGTCTTTGCAGGCAATTTTTGTCCGATACATATGGATGTTTTCTTAAAA**ATATATA**TTT<br>GTTTTCTTCCGTAGTGTTTACCAAATTGATGGTGTTTGACTCAAAGTTGTCAAAGTTCAGTTA<br>ATCTTAAGTTTACTCCCATGTTTGTTTCAGGATATGGCTGGACTAATTAGTAAGATAAATCG<br>CTCCTCCATTGGCCCTTTGCCTCCATGCTACTCTCCATCTATGTAAGTGCACATTCAC**TATA**T<br>CTGTATTGATCATACATGGAGTCTTTGCTTTTTCTCATTTTTCTTGTTCATAAAATCCAATTTT<br>CTTCATCGTCATATCCGTTGTTAGTTTCCTTTTGACAAACTAGCTCTTCCACGTATTGTCTTTC<br>TCACTGTGTTCCAAGTCAAATGATAGA
68	37422578	37423170	593	+	ATTGATCCATCCGTGAATCCATGGTTCCCAATCTCTTACCAAAACTTCAGCAATTTCACAGCA<br>GCAAATCACCCTCGCAGAACTTCAATCTCCAGTACTCCAATGCCCAATCTCCACATCTCCTAC<br>AGGCAACAGCTCCACGCCGGAAGCGATTTCTGCAAGAGCATCGGGGAAAAAACATAAGCG<br>TAAATCGAACAGCACTACGCTTTGCATCAAAATTTAACAAACCATACTCCTGTTCTATCCAAT<br>AGAACCCTCACAGTGAATTTACTCAAACGAGAACAACAACAACAACAATAACAGAAATCCA<br>ACAGAAACACCAAGCCTCACAAAATCCATGGCCGACGTTTTCGCGTCTGTACTACGCACAG<br>GAAAAGAGGGAGAAGCCACCCCTCACGAGAAAAAAAAAAAGAAGTGATTGAGAAGCTTAG<br>CAACTGTACTGCACACAGTTCCCACCAACCGCGCGCCACTGCCCGAGACAGGGACCTTACC<br>GCGGCTCTCCCCACCGACATTCGCCACGATCGGAGTGCTCCGGCTACGACGCCGCCGCCGC<br>CGCCGCTCCACGGCGGGATCCCCGCCGAACCCCGCGACGCC
69	37601434	37601979	546	-	GTAAATGGGGTATGATTCCGTTGTTCCTCTTCCCCGTCCACATCTGGTTCAAGCCTCCTT**TA**<br>**TA**AAAGAGGAATTTTGGAGATTTCTTAGGAAATGAAGCTGTT**TATATAA**AATAC**TATA**AA<br>ATTATCGTGTTCTAAAAAGGTTCTGTTAATCCCATAGGCAACCATCCTAGATCCTCCCACTGT<br>TACAGACTTCCTGTCCTACCACCATCAACCAACAGTAGCACAATTAATTGGTTGAGATTATT<br>CCTTGGTGAACGCTTCAAAACCCATTGGATGGGAATTGATTTCTTCCAATTGAAACTGATTT<br>TTTTTAAAAAAAAAAAACTTAACCTGCAAGTTTTCAAACATTCTCAGTTGAAGTTTGTCATGTT<br>CAAATTGGGTGTGCCTTATCCCAATTGAAAGTTTCTCGGAGATTTTCTCGTGTTCTGAAAAA<br>GTTTTGTTAATCCCATAGGCAACCATCCTAGATCCTCCCACTGTTCCAGACTTCCTGTCCTAC<br>CACCATCATGTTTTTTTTCTTTGAAAAAAAGGGCATCTAATTTCAGCTT
70	37905546	37906115	570	+	TCCCTTGTTGTCAATCATTCAAAGTGAAGCAGCAAACTTAGGATCTCCACAAGGATGCTAGT<br>CAAGGGTATCCTTAAACATTAGTAAGGTCTTAGACTCTTAGTGCACCGTGCTGGCTCGGCA<br>GCAGCAAACAAGAAGGCAATGCATTG**ATATATAA**ACAGAACTGAAAACAGCATATCATTT<br>TTCGAACAAATGAAATGGCATATGATGATTTTAAGCCGCATTGCGCCCAAATTCAACGGGA<br>AGAAGGGAGTACGAAGAGCACGAGCAGACCACTTGCCTTGGCGGCGCTGGCTTTCGCCAC<br>ACCCATCCCGGATTTCTGCGACGGCAACCGTGCCAAGTTCGGCACGCCATCCCATGGCAAC<br>TCCCTGGCATCAATGGGCGCTCTAGAAGCCGGGGCTTCCTGGACCGATGCAGGCTGCTGCC<br>GTTGCTGCTGCTGCTGCTCCTGGATCCTCTTGGCAGCAGCCTCCCTCGCAACCGTCTTCCTCT

					CCTCCTTCCCCACGCCAACGCCAGCGCCGCCACGCTCATGGCCACGGACCACCCGGTCGGG ACCGAGCGTGGTGTCGCTGG
71	38057051	38057650	600	+	GGAGAAGTTGTTAGTGAATACATG**TATATAA**AGGAGACCATAATGCTACCATTGAGAATT AAAAAGAAGATGAATTGCTCTCAAGAATCAAGACCTTCAAGTTTTT**TATA**TTTAATCTAGCT CCTCTCAAAGCAGCAGGTGGCCAAATTTTTTCCTCTCCATCAATCAATCCCAAATTTTTACAC GAACGAACCAACAATTACATGTAGTTCACACTTCGCAGAACAGTGTAGTGTGTTAGGTCCA AGCATCATTCAGCTGCTAAGCACGATTCTACAAG**TATA**ATCTAGGACATTAATGTAAACCA CATCAAAAATCCGCAAATACGCGCAACGCGTTAAAAACTAACAGGGAAATCTCCCAAAATA AGCCGATCCCTGTGTTGGTGTGTAGGAAGGGAGGATTTCGATAGGCCATCGAAAGTGATC GAGCGGGCTCACCATGGGATGTACTTCAGGGAGAAGCCATCTTCGACGCGCGTCTTGACG AGGGCCTCGGGGACCCTCTTACCGAGCTCCCTGAGGACCTCGGCGAGCGGCTTGCTGATG CCTTCGGTGGGCGCGGGCGCGGCCACCTCGCCGCCGGCGAAGTCGTCCTCGT
72	38286122	38286714	593	+	GCCCCAATATTGATGACCAACAGCACAGAATTGCAGTAGGCACATCTATTTGACATCTCTTT CACTAACAAAACAAGAATGCCAC**TATA**AAAATTCTATCTAAATGTACGATAAAAGTGCTAG TGTGCTACAATCCATTCCCACTCTAGAATTTGCACATGTTTAGGCTTCCACTTGTACTCATGT AATACCACTTCAATTGTGGAGTACTATGTAAATGCAAAATGCAACTAGACAGCATTTCGGG TAGGTTAATTCGTCTAAGTACAATTTCTGAGCTGTAGGAGTTAACAAAAGAGGGAGTTTTA ATTCAAGGAGAAAGAGCTGGGAGAAGGTAACCCGTGGGAAAAGAAGGGGGAGCGTGGC CCTCACTGGGATTGGTGATGGCGTGGAGCACGCCGAGCACGAGGAGGGAGTCGCCGCCG GCGCGGAGGATGTCCCCGCGCGTGACCACCGCCCTGAGCGCCATCCTGATCTCCTCCTCCC GGTGGTCCCTCGTCGCGTCGAGCGCCAGCACCACCATCTGCGGCGGCGGAAGCAGCGGCG GCAGGGCCGCGGGCTGGACCGCTGCCCCGCCGCCCGCAGCGGCGCTC
73	38336411	38336924	514	-	CAATGCCCTTGAATGGCTTATTGCTTGCAGGCATGTGCTTGCCTTGCATGATGCTTGGGCTT CTGGCTCACGACCCTTTGGCTTCTTGGGACCCTGGCCTAAGGAATCATTGTCATGATACATG TGGGAGTACTGGATGGGGTTGGAGTTGGAGTGGTTGAGAGGATCAATATGGAGAGAGAA AATAATGTGCACAAAGCCAGATTGAGAAGGTTGGGACGAAAATGGAATGCG**TATA**ATAA GTGATTTTGATCCAACTTGGATTCAGATTTTGTAAGGACTCCCGTGCCGCTATCCCTTGGCG ACATAAGAGGCGAAACAGCCTTGAGCGGAAGCTTCCCCACCGCATCCTGCTCAAGTCGACA TCGGCGATGGCAATGATGGCGTCTTCCACCCTATCCTCCCTTACTCCTTCCCTCTTCCTTTCC CTTCCTCATTTTCTCTGGTTCCACCTTGACTCGCCACCGTTAGCGTGGCCACCAGCGACGAC GGTACCGGCGGCGGCAGCACCTCC
74	38495141	38495665	525	+	AAGGAAACAAAAAC**TATA**TGTGA**TATA**CATTAAGGGATAAATATTGGGTTTGTTGGTTAA ATGAACATTTGGACCTAAATT**TATA**AACAGGGGAAAAAACTACAAGTTATTTACCTTTTGG GGGTAAACATTGATTAAAAAATTAAAATAAACTTAACAGAATTATTGGGGGTAAACATTGA TGCAAAATGCAAAATAATGTTATTTAGCTTTTGGGGATAAACATCGATTAAAAAAATAAAA TAAACTTAACAAAATTTTGGGGTAAACATTGATGCAAAATGCAAAATAAACTTCACAGAAT TAATTGTGGAATAAAACATTGATGCAAAATGCCAAATAAACTTGGGGTAAACATAGATGAA AAAACAAAATAAACTTGGGGGTAACATTGATGAAAAATGCAAAATAAACTTCACTGAATTA ATTGTGGAATAAAAAATGGTCCCGGCGGGGCTCGAACCCGCGACCTTCGGCTCATAAGAC CAACGCTCTAACCGACTGAGCTACGGGACCGCAACAGCT
75	38498507	38499085	579	-	GCTAGAATAGATTCACACTTGCCATTTCGCTGCAAAGATACAGAGCATGCCATCTTGGCCC ATTTCAGGCAGCTTGCTCACACTGCAAGGCTCTCTGAAACTTTACAGCCGCTATCTATTTTG GACACCTACCAGGAAATAACTACAGGATTAAATAGG**ATATATA**GATGCAGGGGTAACAA TTAATCACAACGAGTAAAAAACCATGAACTTGTAATGATTTTTTCATGTGCTTTTTTGAGGG TGAAACCGTAGAATTGAGAATACCCATATGAAATTTTCAATCATGAATTTACCTCAAAAAAT ATTTTTTAAAAAAAATAGGAAAAAATTACCTAAAGATAAATCAATCGTGAAAGCATGCATTTC CCATAGTGTTGCACTGTTCGTCTTGAGCTTGCAGAAAAAGTTGTAACCAGATTTTCAGGACC CTTCATCAGTGCCATTCTTCTTTGCTGCAACAAAACAGCAGGTGACACTT**TATA**TCTTCTCA GGCACATATGGCAAAATCTGCTCCCAACCGTAGGATCAGAGAATGAACGATCCCGATTTAC CCTCTGTCGCCGCTGCCTTTCATTCA
76	38623725	38624255	531	-	AATCATGGCGCTTGGGTTGGTGTGATGGATTGGTAATTGG**TATA**CCTTCTGTGGGTTTACA TAATCTGA**TATA**CCAACTCTGTCTATCGATAATTTCAAAGGTAATTAGAAGCGGTTTTAAA GAGGAAGGAAATTGTGCAG**TATA**ACATCGAGATTCGTTTTATTACAGTATTGGCAGATTTT GTATCACTCTGTTAGGAATAATCAATTGGG**TATA**CCCTTCTTGATTATTTAGTGTTTAAATTT GGAACAAAAGCAACGTCGTATTATTTGAGCTTAGC**TATA**CATTGTCAAAAAACTCGGAAGT GCAAGCT**TATA**TTGTGATCATGCAAAGTTTAGTAAAGTCAATATCTGATTCCTGTACTATGT ATTGCAGATCGTCCTTTTTTCACAAAATAGATCCTTGGATTTCTTGAAACATGGTATCTCCGG

					GTGAAATGTCAGTAAGCAATCCATTACGCTTCCAAGGCTTTCCTTGTAATGTGGTAAGCGA<br>CCTTTGCACAACCGCGGATGGACTTTTTTCCCCCATAA
77	38642005	38642535	531	+	TGCTGTAGAAACGGAAAAGCCCCGCGCCATGCAACCCTCGTGTCAGCAACGCACGGATCAT<br>ACTAGCGAAACAAGCACAGATACCGCCTCGATCCGCGACCGCGGTAACAGTGCACGGCTG<br>CACGAACGCACCCGGCCCACCCCAACCAAACGGACCGGAAACAAGAAAACACGGCAGCTA<br>GCTAGCAAACCATGCAGTTTTGCAACGGGCAAAATACGAGAAAATCCGCGACAATGTGTG<br>GTTGCCGCAATCAACCGTCAAATCCACGCTGTTTTGCGTTGCCGCATCGCGAATTCAGTTGG<br>TTTAGCTCAAAATGGGACGAGATGGTACTCATGGTAGTAGTAGAG**ATTATA**TTCAACTCT<br>ACTGCCGCACCAAAAAAATAATCTCACACGAATTCAATGAAAAAGTTTTGAGGAAAAAAAA<br>TTACAATGAGTCATTTCAGGACCCACCACTAACAATGCAACTATCTTCTCCTCGCTCCGTTCC<br>TTCCGCCGCCGCCGCCGGCGGTGGCGGCGGCGGCTCCGCCG
78	38839961	38840463	503	+	CCGGAGTCGCCATCGTCGCCGTGCCCCCCGCGCTCGCCATCTTCAACGATGGGGAGGAG<br>ACCGAGAGCTTGTCCTGATTTGGGGGGAGATAATTTGGATTTGGAGGGGAGAGGATCAGA<br>GGAGAGGAGGGAGGGGTTCAAATAGGGGCGGCGCGATGCGGCGAGCCGGCGCCGTCGC<br>GGAGCCGAGGCGAGGCGGCGTCGCGTTGAGCCGTTACGCGTGTTTTTTTTTTTTTTTCATTC<br>TTCTTCTATTTTTTATTTATTTTTTAATGTCTAGTGGGCAAGTGGGCAACGACGAGTCCAGCC<br>AGAATCCCATCCCCTTTCAGCCCTCGTCGCCGCGGCGGCGGGCCGCGCCGTCGTCCACCGC<br>TGCTGGCGAGGGGGCGGCGGATGAGGCAGGGTGGTCCAGATCACGGGGCCTCCACTCCA<br>TCGTCTCCCAATCCCTCCCGGAGCGCGCTCCCCGCAGCCGCAGCCGCCGATGTGGCGGCCC<br>GGCGCTCGCCATGGTCGTG
79	39017588	39018179	592	-	CCACATTATTAAACAGCCCAAAAAACGGACAAAGAATGAGAACAAAACCAAGCAAGCCAT<br>CAACCACCACCTCTCTCCCTCTCCCTAATTAATATCTTCACCCTTCACTAATCACCTACCCTCA<br>TAGTGACCCTTACTTAGCTAATTGCCTAATCACAATTAGAAAAAGAAACAAAAAAAGCAAA<br>AAAAAAATCATCAAAAAGCAGCCAAAATACTCCTCCTCCATGTAAGCTGTATCCACCACGAC<br>GACGTCCACTCCTCTTCTTCCTCCTCTCCTCCTCTCTCTCTCTTCTTTCTCTCTCCTCGCGAGAT<br>CTGTGGCGTCACTGACCTAGCTTGTTCTTGCTAGCTGCTGCTGCTGCTTCGGCGGTGGCGG<br>TGTGTGTGGCAATGCCGTCTCTCCGGCGATTCTTGGAGTTGGTGGTGGTTGGGATTGTTGT<br>GTGTGGTGTCAATGGCGGGAGTGATGGTTTGGAGGTTGCGGTGGCGAGGAGGCAGCTGC<br>ACCAGCCGTTCTTCCCGGATCAGTCGTCGTCGCCGCCGACGCCGGCGCCGCCCGGCCCCGC<br>TCCGCCGTTCTTCCCGGCGCTGCCGGTGCCGCCGCCGC
80	39042251	39042840	590	-	GGGGGGCAAGTAAAAAAAATACAAACCTTGCAAGTTATCAGTTTTAAAACGCTGAGGATG<br>AAGCATCCCGGCTTGTAACTTGCATATTGGTCACACACTCACCGGTCAAATTAACAGCCAAC<br>AATCAAC**TATA**TCCATTAACTTGATGTTCTAACTACGGAG**TATA**TCAGAGAAAACATGCTT<br>CTATTAGGAAAAAATAAGCACTGAAAAATACATATTGCATTATTATCGACTGCA**ATATAA**T<br>AATGATGCACTAACGAACTTGATGGATATGGAAGGGAATGCTTTTTTTACTAATGACCAAA<br>ATATCCCTCCTTGCAATAAATGGCAAACGATTCAAAATGGAGCTAACGGGGGCAGTTTAGT<br>AATAACTTTCACCATCACCGCAATCCAGCAATACGCTGGAAACGTGAAGATTGCAAATAAT<br>TTCCATTTGCCACCCTACTTATCCCTTATTTACCAAACGACCCCTCGTCTTCCACCTCGAGCC<br>GCGTCATCCAAAACCGCAAAATCGCGCCTCTCCGCCATTCCCAAACCCCTCGCCTCGGCTCG<br>CCTCTCCCCATCCGGAGCTCCTTCTCCGCACACGGCCA
81	39124688	39125264	577	-	AAACTAATTCTTATCTATTTCTGAAGGAATAAAAAAAATACTAGAATTCTTAATTTTTCAAAA<br>ATTTTCTCATTGAAACAATCAAAAAATAAGAATAGGTTTTGTTGGTTAAAGTCAAAAAGTTA<br>ATGAAATAACTTCGTTACCTAGTTATTACCTAAAGAAGGACTTTTATTAAAA**TACAAAA**AA<br>AAGATTGAATCATTTTACTTTAATATTTTTTTGTATTAAAATGAAGCAGCTCCCTTGTTTCGT<br>AACTCAAATTGATTGGAATTCAACCCTGCTATGATCCCTCCTGCCGAGAAGGGTGGAAGGA<br>CAGGGGAGCACGCCTGTGGTTGAGCTAGTGCTTCATACCTTACTGGACAAGGAAGGAAAG<br>GCAAGGAGGATCAGTACGGGAGAGAAAGTGATTTAAGAATGTGAATTTGATTTGATAAGT<br>AATCAAGGCAATTAGCTTAGGTTCATCCCGAATCCATTCCATGTTTCTTAAACAAGTCCTTCA<br>AACCATCGTGCTAACAAGCCATTCTCCTCCGAAAGTCCTAGAGCTTTTTCTGGTCCATATGC<br>AGCCTTGAAGAATCGAGATGCC
82	39160475	39160994	520	-	TAGTCAAGTTCTAAAATATGTCTTCTTATTTTCG**TATAAAGATT**GAACGGACATCAAAGTA<br>AGCAAGCACAAATTATCCAAGTGCTAAAGATAAAAGCAACACTGACAAAACATGTGCAGC<br>ATAATCTCTTTTTTTTTTGTGCAACTAGAAGAGCATAGCCCCCCCCCCCCAAAAAAAAAA<br>AAAAATCCATTAGCAGAAATGAGCATTACAAAGTTTTCACAACATAATCTAATTATGTTTTG<br>CAAAAGCATGTAACAAAGCACCACATATTTACCTAGCACGGGTCCATGGTGTCATAACACC<br>CTGCCGATTGAGCCAAACACATGGAAACATAAAAGGATTCCTTCCAAAATCAAGAAGTGGT<br>CCCTGCAAGGAGAACATGTAAAAAATGTACTGGCAAGACTGAAAATGACAGACCAAGTAA<br>ATGAGTCAGGGAATAACTG**TATA**GGCAGCTTCAGCAAATGGATGAAAGAGAACAGCCTCT<br>TTAATGAGATCCTGAGGAGCCTCTGCTTGTTCT

83	39174471	39175054	584	+	CCACGGCTAAGATTTGCTCAAGATGGATTGGGAAGGTCAAATGGAACATTGTAAAAAAT<br>ACAATAAAAAAAAAAGTCAATCCTCACTATGAAACATGTGTCCAGATTCGTATCTATGATTGA<br>GTTTTTTAATGAGATGGATATGGTACATTTACATACGCACGAATACATTTGCACGCAAATAT<br>GCAGCACCAACTTGAAGAATTATCTTAAAGAAAAAAAAACCCAACACTTCCCCCCCCCCTCC<br>CCCCAAAAAAAAAAGGCTTGAATAAAAGGAATAAAAGAAAATGTGGACTTTTGGAATGATC<br>AAGGCATCTCCTAATTAAATGGCAGAAAAACACAGTGACACTGACACTGAGGTCGGTCGTT<br>GACCACCCCCACCCCCACCCCCAAAACGGCCAGCATCAAGGGTCGCTACACGTCCATCCAT<br>CCATCCATCCATCCATCCACGCGGTTGACGCCGACGGCCGCGATCCTTCCACGACCACCCCT<br>TCCCCACCCAATTAAATCCTCAAAGCAACCGCAACCGCGCGGCCGCCGGCCACGTCGCG<br>CCGCGCCGCGCCGCGCGCCCCGCGGCGTGGCCT
84	39187869	39188419	551	+	AGCAACAATTTGGATGGCATGGTTGCGTGCAGCCG**ATATATA**TCTACGAGACTATCATCA<br>AGGCAGAGCATGATGCAATCTGATCTGCTGACGAATTAATATGAGCAACCGATCATAATAT<br>TTTTGGAGTCGTTTTCAGGGGTGTTCATGGGGAGGATCGAGGACAGTGGCCGGGGGAGG<br>GGATGATGATTTGGATGGACGATAATCCGCCATGTCGACCTGCCTAGCTCCCGGAGCTCTT<br>CAATTCGATCGATCGATCGGCTCACTGTAGCCGACGTAGTCCATCTCGATCGATCCCTAGCT<br>AGCTCTCCATCTCCAAGGCTTAATTAGCTGACTATGTTGCCACTTGACACTGTCACCCCCTAT<br>CTCTTTCTCTCTTTCATTGGTAATTAACTGGGGGAGAGTGATAGAGGCAGCAGAGCCAGAT<br>GCTGATCGAGATTAATTTGACACTGATCTCTCTCTCTCTCTCTCTCTCTCTCTCTCTCTCTCT<br>CTCTCTCTCTCTCTCTCTCTCTCTCTCTCTCTCTCTCTCTCTCTCTCTCTCTCTCTG
85	39320792	39321360	569	-	ACGTCT**TATA**ACTTAAAATGGGAGTAGTAATATTCATCATTCATTGTTCTCTGTGTACGCCA<br>TTTAGGTTTGTGTAATATGTTCTCTGTCATGTTGCACTTTGGCAGAAAAGGAGCACGATACA<br>AGTGTATGCTGTTAGATGAGCTAATTAAACAATAAGAACCTACACAAAATTGTAAAACAAC<br>AAGCCTCTAATCAGTGGGCTTATT**TATA**CAAGTGTTCATGCTCACACAACGGCTTGAACAG<br>CATCGTGTTTGCCCATCATCCATGAACGGACAATGGTGCTCGATCGAGAGCTCTTTATTTAC<br>GTCCAGCATCGGTGTCTACCTCTCTCCGGCGGCCGGCCGGCCACCGGCCACCGGATGCCGG<br>CGTTCTAGCATTTCCAGCACGAGGCGGCGATGACCGGCTTCCCCTTCCAGCCGAGCACGAG<br>GCAGCCTTTCTCCTCCGCCACCGTGTACCCGCCGCCGCCGGCGTTCTCGTCCAGCCACTCCC<br>GCGCCTTGGCCGCCATCTTGATCGGCACCGACTGGAACCCCGCGCGGCTCATGCGGCGGC<br>GCCACTGGTCGGCGCGC
86	39346711	39347231	521	-	TAATAATAAAATATTGCGTATTAATGCTACTCTAAAGGAAATATCAAAGATCAAAG**ATTAT**<br>**A**TGTTTTAGAATTTAATTTATTGTCACTTAATGATAAAATGTTGCCTATTAATGCTACTCTAA<br>AAGAAATATTAAAGATCATATGTTTTAGAATTTAACTTGTACTGTTCTACAGGCTGTGAGCA<br>TTACCAATGCTATTTTCGCTGTTATTCTGCTTGGCATGCTTATCAAAAAGTACAAGAGCTAC<br>AAGGCTTACAGACAAGAGTATCGCTCTCTTTCCTCGTCATTAGCGAATGTGTAATTTGAGTA<br>AGTCAATGCCAGCTATGAGAGCACAAGCATGCATTAATTTTGTTTTGTGGGATGTTGGTAC<br>TCAAAATCGATTAGCTATTTGATCCGTGGAATCAATCTTGCATGTATGCGTTCAGACTTTAG<br>AATTCAGATACTGGGCGTGTAGGTGTAGCTGTAATCAACAGCTGTATTCTTTCTTCGCTGGT<br>CGTTATTGCCCGGCGAGCATTTGCCT
87	39632140	39632733	594	-	ATACAAGAAAATACCAAGCTAATGTACTG**TATA**TCACAGTTTGAGACTCTTGTTTTAGTCCT<br>CTAAACAAGATGAAACAATAATGAAGAACCAGCCAAATAAACAAAGTAGCTAGGTGG**ATT**<br>**ATA**CTTAGTAGTTGATGCTTTCTAAAAGAAAATGAAGTTAAAAGACTCACTAAGCACTAGT<br>TTTGGTTGTCTTGGCCATTGTCACTGTTGCTTACTTGCTTTCCCTATTGCCTGGGAACAATCG<br>CAGACGCACGGCGACCAGCTTCGCTGTTGCCGTGTCTCGAGCAGCCGCCTCCTTGTTGTCCT<br>TGTCGGCTGCCATTGCTACAATCAGGCCCACCTACACGGCTTGACGAGCTAGTCAGCTAGG<br>TGCTCCTCTTCCTGC**TATA**GATCAAAGCTGAGGTAATGACCAGTCGTCCATCGTCACATGC<br>GCCACCTATGATTTGCTCCGCCGCGCGCCATTTGACGCCTACCTGAGCCACCAATCCGCCGG<br>CAACCTCAAACACGCCAAGCTCTAATTAATCAAGCAGCTAGCAGCCAATGCCCACCACTCCA<br>TCAGCCCTAGCACGACGAATCGGTGATGGGGAATTGGGGA
88	39641227	39641815	589	+	GAATTTTGGATAATCTGCTTCAACCTTCAATGTTTCATTGCTTCTTGCACCTTATTCTTGTTGA<br>GGTGATACCGTATTTAACCATTTGTACTACTGTCCTGAGTCCTGTGGAAACAGTGGTGATTC<br>AACCAAGGAACCAAAGC**TATA**AAGGTACATTTTCTCTTACCAACTTATTTTTGTATTGCACT<br>ATTGCCAACTTAAAAGTTGACAATTTTA**ATTATA**CTAGCTTAACTGAC**TATA**TGATTATTTT<br>TCCATTGCATTGCTGGCATGCTGCTTGTTGATGATGAGCTTCTTCACTGAGCTGAAGGCCTA<br>AGCCCTGGAATTGGAGGTGCAAGTTGCTATGTTCAGCCTTTGGCATGTTGGCACCATCAAA<br>CAAAAGGTA**ATATAA**AATATTCAAATCCTAAACACATGTATTTTCTCCAAGTTGGCTGTTG<br>GCATGCTGCAATTTGCTATGT**TATATAA**ATGAAATTGATAAATTGGCTTAGCCATTGGATA<br>CAAGTTGCCTGCTGACTCCTCCCTCCCTAGTCAGACCTAGGCGCCACCTAGTTGGCCCTAGG<br>CGCTAGGCACCACCCTCCCGCCCAACTAGCGT

89	39707555	39708142	588	+	GACCTAAACGTCTAACGACCTTTTCAGTTTTCACCACAACAACAATGTGATCAGGTACAGTT<br>GTATTGTGGACAGAAATACAGAATGCATGCTTGAAAATGCAGGATTACCAACTTCTTTGGT<br>TTTTCCGATGAATGCAGCATCACCTACTTGTTGGCTTCTCTAATGATCGCGTTCACGACTTAA<br>GCACAGTTTTAACAAAGCAAAGAAGCAATAACTGACCTAAACACTCCGGAAATCAAACACA<br>GAACAACCAGAAGCTTAGCGTTCCGGTAGAACACAGCCCCTCCGTTCTGCGCCGAGAAGAT<br>GGACGCTGCGAGGAGATGGGGTATCGCGATCTCCGCGAGCTGCAACCACAACCACACGTC<br>ACCGCGTTTTAGACCCATCAACGAGAACTCTGCAGCAGCAGAGGTCAAACCCAGAAATCGC<br>CATTACCGCAGCGCCGACGAGGGACGCGAATCCGACGAAGACAACCCATCGGTCGGAGGC<br>GACGAGCGCCCACATCCGCCTGAGCGCGGAGGCGGCCGTGGCGCCGCTGGCCTCGGCGC<br>GGCCGCCCCCGGCCTCCTCGCCGTCCTCGAGGCGCCACCA
90	39708313	39708880	568	-	TGTTCCAATGCCACGACCACGCACGAAGCCGTTCTTTCAATCCTTCTCCGTGTCCAATCCTAA<br>AATCCAACGACGATTATTCTCCTCCAGAAAATTGTTTTAAATCCCCCAAAATACCACCACCAC<br>ACCACATCGCCTCATTCCATCCATTTCTTCTCGTAGAAAAAATCGCAAAAAAAAAACACGTT<br>AGGAAAAAGAAAAAAAAAAGAAGCACACGCGAAAAGTGGAAGAGAAAAAGGATTAAAA<br>AAGCCGCGGAAAAAATACAATTTAGCCGGAGAAGGTAGAGACGTTACGGCGCCGGTGCC<br>GCGCACGGCAGAGCAACCAACTCCGGCGAGGACGCAGCGGCCTACGCCTATTTAAACCTC<br>GCCGGTGCTCGCCGAGGCGGCGGACTCTCTCACCGGCGAACGCGGAATCGGAATCTGAAT<br>CGGGCGGTGGGGGTGGAGGGGAGGGATAGAGGGGGTTTGACGAGAGTGGAGAATGCC<br>GACTCCGGCGGCGCTGCTCCTCACCGCGGCCACCGGCTCGGCGGCGCTGTCGGTGGGCGT<br>GGCGGCGGTGGGGCCCAGGGCGCCGT
91	39773082	39773671	590	-	TCCTAAAAACACCAAATAACAGAGACATCACATCAAAATATTGTTCACAAAACTAGAACTAC<br>AATTAAGCACATTAGCATAAGCTCGATAGGAACGGTCACCTCCATAGCCTCCCGGAGAGCG<br>CGCTCCACCAGCTCCTCCTCTGAGAGGACCCTCAACTCATCTACGCTGCAGAATCCACAAAA<br>TGTACAAACCAGAACACGAATAAGCATATGAACAACATGACAGACACTGGGGGATTTCTCC<br>ACCTGATACGGCGGAATTTGGGGGGCTTACCAGAGCTCTTCGTCGAATTCATCACCAGGAT<br>CGCCCAATTCATCTTTCAGCGACTGCTCGCAAAGTAACACAGAACGCGATTACACCAAAGC<br>AAGGGCACGGATTGAAGAGCTAGGGTTTGGCGGTGTGCTCAAGGGGATTCGAACCTGCA<br>GCTCGTGGAGCGCGGAGGACATGAACTCGGGGACCGTGGAGACGGGTCCGACCATGAAG<br>GGGACGAACACGGGCCCGCCACGAGCGAAGGGCATGCGCCGCTGCTCGCCGGCGGCAGA<br>GCTCTCCGCCTCCTCCCCCGCCGCCGCCGCCGCCATCGCCGTGTG
92	39783698	39784289	592	-	GGTTGGTATTTTTAATTCGTAGCCATTCGATCCAGCCACAACCTACGTAATAATTGGT**TATA**<br>GCTTCTTTGTAGCATATGTTGGGATGAATTTCATTTTAAAAAATGTGTATTGCTAGATTCCA<br>AGAGCATAAAAATTTGCGAAAATTAGGTCACCTAAAAATTACACATTCATAAGCCTAAAAC<br>TGCATCGGTATGCAA**TATA**CAATTTTACTTATTTTTAAC**TATA**ATATTATTCT**TATATATA**G<br>TTTTGATTTATTAATTTGTATCTGAGCTTGAGTACTTCTAAAAAAAAATTAAATCTTTATCTTT<br>CATGTAGTTTGGTAATATTTATTTTTTAGGTTAGAATAGTTTACAAAGTAGCGTGCCTCGC**T**<br>**ATA**AGACATGTCACGTGTGTTTGTCATCTT**TATATAT**GATATCCT**TATA**ACATGCTTTAAG<br>GCATGT**TATA**CTTGAGTATGAAGTATGAATCTTTTCACTCCCCCCCCCCTCTCTTCTTATTCC<br>CCTTTCCCTTTTAAAAGTTGCCAACTATGCAAGGTCAATTAGCAGCACTTCTCTCACGCATAT<br>GCAAGTAGCTAGTAGTGCATGTGCATGGGCGA
93	39903842	39904399	558	-	CAGAATGGCTAAAAAAGACTTTTAACATGC**ATTATA**TGTTTGATGAGATTGTGTGATGAAC<br>AAGTATGGAAAAGTAAGGTGAATGAAATTATTTTTTGTTAAAAACAATGATGCAACCAGCA<br>TTTCCTTTGAAAACAGGATACTCAGCATTTTGGCTTATGCAAATCTTTGTGTGCCGGCTTTG<br>GATCAACAGAGAGAACTTTACAGAGAGGTGATCTTATGGTCCTCAACCTTGTGCGCATGGC<br>TTTTACAAAGCACACACACCAAACAATGGTACAAAGGAATTTTTGTATGTGGTCTCGTCTCC<br>ACTCCAATCAAAGGTTAAACTAAAAAGGCCATGAATGACACAGACAGAGAAGAGTACAGC<br>AAGTAAAAAGTTTTAATGCCATTAGCTTAGTTAATTAATGAAGAGCCATGAATGAGAGAGA<br>AAGTGTGAAAAGTTTAACTAGTACTAGTAGTAGCTTAGTTAATTAATGAATAATTATTTGGA<br>GCTTAAGAAGGGGGCCAGGAGAGACTGGCCTGTTCACTGGTTGCAGTCCTTCCATGCACA<br>GGGAGATG
94	40125259	40125760	502	-	TGCTTATCTTTATTGGATCAGAAAGCTATCTCCACCACTGAAGCCCAAGTCACCTTAGAAAA<br>GG**ATATAT**GTGCCTCTGAATAAAAAGGCAAATGAAATGATGGATATGTGGAAAAACACC<br>AACTAGCATAACTGTATCTGCTAACCAAAACATGCTATTATTGTCACCAAACTACAATAACT<br>TTTGTGGGGTTCTGC**ATATAT**CAGCGTTTTATTTATTGTTTGATTAAATTTTGTATGTGTTCT<br>TGAGAAATTAATGCTTTTTTTCATATTGAAAATCAATCAAGTTAACTGCATCCCAAAGGATT<br>TTTTTGGGGGCAATTTTCAAGAGAGTGGAAGGGAACCATATTGCTAAATTAAACCATTAAA<br>ACAAGGAAAAAATTTATCTGAAAAGTTAGAGTTGGAAGGATGATGGCAAGGTATCCACTC<br>GATTTGGATGACATTCTTTTGAGTACAAACT**TATA**AAGCATGCAACTATA**AATGCTTCCAC**<br>TTGCATATGACT

95	40174512	40175083	572	-	GCCAACATAGACGGAAGTTAGTTTTTCTATGTCTCTGGATTGGTGTTTTACATAGTTGTCTT<br>ATTTTTAATCATATTATGGTTTTTTTAGTT**TATA**GTATTTTATCATGCAAGGCTGCAACCCCT<br>GTAATAGTTGTGGCTATTATTAAAAAACTAGCATCAAAACAAACTGCATTAGGATGGGTTT<br>AAATTTGCCCAACTGTTTTCTAGGCCACTCATGAGAAATC**TATA**GATTAGCTTTATTTTAAG<br>GAAAAAATTC**TATATAA**AATTACATGTTAGTTTAATTCAATCACAAGGAAACATTCCAAAA<br>GGGATGATCTAACCACCCTTTGAATTTTTTTCTCTGAAACATTTTTTTTCAACCATTGGATCA<br>AAATCTAGCCATTTACATTATTCCCCATACCACTAATAGCTAGGAGAAAAATAACCACGTGC<br>TATCATCGTCGCCGCCACCCGATTCACATTGCACTTAGTCTTCCCATCGCCTCCACCTAGGTT<br>TACATTGCTCCTATCCCCTCGCCTTCACTGCCGACCGCCGCCTCTCGATAACTCTCTCCTTGTT<br>TCCTCCACCGC
96	40251423	40251940	518	-	TACTCATGGTTCAATCTCTTGGCAATGATGAATCTGCACAAGATTTGCAGTGGCTGACAGG<br>TCTTGCAAAATCATCTACTGTCAAGCCATTGCATATCAAAGAAAAAAGTAGTGCTAGTTTTT<br>TTCTTTTTTTAAAAAAAGAAGAAGAAAAAGAATCAGAAATACTAGTGGGAGGAGTACAAG<br>GAATCCAAGGCTGAAAAGAGGCCAAGAAAAGCATCATAAAATGCTAGC**TATA**TTTCAGCT<br>CACACTATCTTCTGCACACTTCCCCTTTGAAACCAAGCAGGAAAAAAAGCTTGTCAGAAAG<br>GAAACGGGTAGAAAGAAATTAAAGAAGTTTGAGAGAGTGATGAAAGGATCAATTTGGTA<br>AAAATAAAAAGAGAAAGACATTTCAGATTCGATTCCAACATGACACAATGGGTAGAGCAA<br>AAGGCCAAAAGAAAAGGCAGGAGTAGTAGTAGAAAGACAGCAAAAAAAAAAAATTCTTGG<br>ATTCAGCAAAGAGATAAGGCAATGGGGGTGAGAAAG
97	40253694	40254174	481	-	TTGCATGTTATTAGTAGCAGTTAGTTCCCTCATCACCCATCAAGGATCAAGAATACTTCCATT<br>AATTTCATGTTAGAGT**TATA**CCCTAATGGCTTAATTACAAGCTGTAAAATCTTCAACCTAGT<br>GAGTTGAGAAAAATACTTTGAGATCCGTAGGGTAAATTAATGCGATATGATCATCATTAGT<br>TCGTGCGATGAAGCTGGGAAAAGAGAAAAGAGAGATGGGGATGCAAAGAAAGAACAAG<br>AAGCAGCTGCATACACTGGAATAAAGCTGAAAAGAAAGAGGC**TATA**TGCTCATGCTCCTA<br>CTACTGCTACTCCTCTCGCCTTTCTTTCTCTCCTTTTCAAGCTCTGATTTCTACCTCTCTCTCTC<br>TCTCTCTCTCTCTCTCTCTCTCTCTCTCTCTCTCTCTCTCTCTCTTCTTTTTCTTCCCCCC<br>TGATTCCTGTGCATTTGCAGGTTTGTTGCAGGCAAAAGCTCGCGT
98	40268982	40269562	581	-	TCTCGACAATGTCAGGCTCTGATCCTGAAACTTTTTGAAACATCCATTTGCTTGCTGGTCAA<br>AAGGTCCTTCCACTCAAATCACACCAGCTGACAGTTTTTCTTTTCGAGAGTTCCTGAGTTCTC<br>GAGTGCTGGGTATCCTCAACAGCGCGTGCATTTGACTTCATTGCAAATCCTCTTGCTTATTC<br>CTTCTGCCAAGCATCTTCTTGTTCGTGCTTCTCCATAAATCTAGACAACCTAAGCCTGAACTT<br>TTTCTTGGATAATGAAATAAACCTAAGCATGATCATGCATCTCCGTTCTTTATCAGACAAAA<br>ACTTTTTCTTGAGTTTCTTGTCGTAATTCCTCTCCTTAATCCAGCTCATTTTGATCCAGCACCA<br>TTTTGTGGCTGACAGTGTGCATCTCTTGTGTTGCAGCTGAAACACGCAATTAAGATCAACTC<br>AACTGCAGCAACTGCTATTAACAAAGGGGCTCTTACTCGTCTAATCATGGTCGTCACGTCTT<br>CCCCTGATGCATCCATCCGCTTCATTATTTTACAGGGGACGTGACAGCATTGGCGGTGCCG<br>GTGTTCGCTGCTCTGCGGCG
99	40383410	40383964	555	+	AGGCTAAAACTTGCCATGTTTTTGAACCATGCTTTTTGAATGATTTTCTTCGGACGCATAGA<br>ATGATTTTTTTGTCCTACTTCTCTTCGTCTACAAATTCAAACCAAATCTAACCGAGTAATGTG<br>ATGTACTAAAGCTAGGTTTGAGATTTGAGAAGTGAGAAAGGAAGAAGAAAGACAACAAA<br>AAAATACTGACCTTCCACTCCCTGACTCCCGCACAACCGCAAGCCGACGCCGCACGCCGTTC<br>ATCGCCGCCGCACGGCCCGCACGCCTGCCTGCCGCCGCCGCCTCGCGCAGCCGTACCCCTG<br>TGACCCCGCCGCTGCGCTGCACACCCGCAAGCCGTCGCCACCGCCTCGCGCCGCCGCACCG<br>CACGCCGCCTCGCGTAGCCCCGCCGCTGCCGCCTCGCGCCGCTGCGCCCCACCGCCGTACC<br>GCACGCCGCCTCGCGTAGCCCCGCCGCCGCCGCCTCGCGCAGCTGTGCCCCTGCCGCCGCA<br>CGCCGCCTCGCGCCGCCCTGCCCCGCGGCCGCCGCCCTATCGAGCGCCCAGCGCCGCCGCG<br>CCCG
100	40581730	40582311	582	-	TGGGTATTGAAAACACTAGTTTTT**TATA**GGTTTTTTTAGACAAGGTGGGTTTTTTTTTCCGT<br>GGGAAGTACGATCGGGGAGAGGAGAAGGAGGACGACTCCAATTTCGTTGTGGTAAAGAA<br>AAAAACTCAATAGCTAAATAGCTGGATGATTTTTTTCCGCTATTCAAGAGTACACACAAATG<br>AATTAACTTTCCTAAAGCTTAAAAAATACATTTAAACCAACTTTTTCAAGAAATTCA**TACAT**<br>**AAA**GTTTTGAAAAGAATGTCGTTTAGAAGCTTGGAAAACGTGTTGCATTAATCCACTCTAA<br>CAAAAATATCAAAAAGAACGGGCCCTATCTCTGAAAAATACC**ATATAA**GTCAGCAAATAT<br>TGCAGCAAGGAACAAAACAAATTAGAGTTCGGTTGGTTTGGAGGCGAGCTAGCTGCAAAG<br>CCTGCAAGTTGCAGCAACAATCAGCAGGGAGATCTTTTCTCGCACAACTTGATCTTAAACCT<br>CTTCCTCCTTTTGATTGATTTTTTTTCTTTTCAACGCACTCCCCTGTTGATCCTTTGACTAATA<br>**ATATAA**TTGTCTTAGCACAGATCAGCTGGC
101	40589635	40590232	598	-	CCTTTCTGAAACCGAGTCAACAAAATCACGAAAAGAATCGTCCAAATCTGCTTATTTTGCTC<br>AGGAAGAACATAACTTCCTTACACACTAATTTCATTCTCAAGCTTATTATCCTCAACCATTTC

					ACAAAAGCCGAGCCTTTCTTCTTCTGAAAATTGGGTACAAATTTCAGTGTTTGCGCAATCAA<br>ATTCGTCATTTCGCTCAGAATTTTTTT**TATATAA**AAAGCTTCTTTTTGGTTGGAGAAAAAAG<br>AAATTCATGAAAAGAACCATACAAATCTTGCTGCATTTTGCTCATAGAGAAAAGATTCAAAT<br>GTGCAGATAGGGTCGGGGACGTACAGCAACGTGTACAAGGCGAAGGAGGTGGAGAGCG<br>GGCGGGTGGTGGCGCTGAAGAAGGTGAGGGTGGATGGCGTGGGCGAGGCGGAGAGCGC<br>GCGGTTCATGGCGCGGGAGATCGCCCTCCTCCGCCGCCTCGGCGACCACCCCAACATCGTC<br>CGCCTCCGCGGCCTCGTCACCTCCCGCCTCGCCACCGCACCTTCCCTCTACCTCGTCTTCGAC<br>TACATGGACCACGACCTCACCGGCCTCGCCGCCGCCGCCCTCGCCGC
102	40591061	40591638	578	-	ATCATTTGTCCATCACTGCAAGCCTGCAATATGATATCCTACCAAATACTCCATCCGTCCCTA<br>AAATACCAACCTATTAACAAACATAATATTTTCTAAGACAACAAATT**TATA**AAGAAACCTGT<br>TCAGATTT**ATTATA**T**TATA**TGAAATATCACATCCAATATTAAATTAATTTTTTTAACGGAGA<br>GAGTACTACCTGACAAAAAAAAACATTCTAGTCTCCTACCAAAA**ATATAA**TAATAAATAAA<br>AGATTTAAAA**ATATAA**TAATTAATTAAATAATTAAAAAAACAAGCGAG**TATA**AATGAAGT<br>AAAACGGCGAGTCGAGAGTCCAAACTGCCAAACCGCCGTAGCACGAGAGACAGTACACGA<br>GACCTTCCCGCGTCCTCCTCCTCCGGCCACGTCGACGTCGCACCAACCGCCGCCGCCGCCGC<br>CGCCGGCGACCTTCACATGACGCGCCGCCATGGGATGCATCCTCGGTAAGCTCGCCGCCGC<br>GCCAGGCTCCTCCCTCTTCTTCCCCGCGGCCGCCGCTGCCTCCGGCGCCGGAGGAGGAGGA<br>GGAGCGGGCGAGGTGCAGCTCGCGGC
103	40601295	40601872	578	-	AACCAAGCTGCATGTGCTCCAATAGTAATTAACAATTGCAAGTATTTTGTTGGAGAGATTCT<br>TTAGAAGACAGTGTGTTTAATTTC**ATATAT**CTGAAAGAAGCACAGTTTGTTTTTGTTGCAA<br>ACGATTGGTTTTAGTTAACCTAATTTTGACTAATCTAGCTCACTCCATCAACAATTAGAACAC<br>**TATA**GCTAGTGTGTTAATCCATACATCTAAGAATTAAACGAGATGGAATCAATTTGCAGCT<br>GAGTTTGTCGTATCAATTACCTCTCCTTGCGCTTCTGGAAGAAGCGCACCAACGATCTCTTG<br>TTCGCAATGGGCAACTCTGCACAAACAGGCGAAGAAATTAGGATCATTATCGCTTAATTAA<br>TCCACCTTTTTAATTAAATCTGAAGACATGCATGG**ATATAA**ACTTAAGCAGACGATCGATC<br>GACCTAGCTAGCTTCTAGCTACCTACTTACCTCGCTTTAGCTTCTCCAGCAGCTGGTCGCCG<br>CTGCTGCTTGCCGATATCGCCGCCGCCGCCGTCAACTGCCGCCGGCCGCCGCCGCCGCCGT<br>CCTCCTCCTCCTCGCCGGCTGCCA
104	40637308	40637889	582	-	AACTCAGC**ATATAT**GTCAAAACATGCGACGACTTTGCCTTTTTTATTTTCCTCAACAAACAT<br>GTGATAAATCTCAAACTGAAATT**TATA**TT**TATA**TTACTACATTG**TATA**TTGCGAGTAGCTT<br>GTTGTCGTCTTTATTTTTTTTCCCAGCTGTCCGCATCAGTTACTCCAGGTCAATGGGTGTAAA<br>AAAATACTAGTGCTCCTTGTTGTCGTTGAAAGTCAACGGGTGTATCAATTTTTTTTTAAGAG<br>AAGGGTTGGGTGTATCATG**TATA**GTAGTATG**TATA**AAATATCTGTAAAAATAGTTTAATT<br>ATGCTGTAACACATTCGATTCGACGACAAAAACCGCTCAATTTAATTCTACTGTAACGTGGA<br>TAATTATGCGGACAAAACGAATACGGAATACGCCTGCACCGAATACGCTTTCGCCGTGTCG<br>AAGCGTAATTCTACTGTACGTGGTACGCCCCAACGGCAGAGGATTTCACATTTTCACCGCCT<br>TTTCCTCGTCGGCGGCGGCGACCGACTCCGAGGCGTGCCTCCCCTGCGGCGGCGACGATG<br>CCCTGCTCCGGCGAGGGTGCGCGCGCCGC
105	41021900	41022493	594	-	TTGCATGCATGTAATTTCCCATTATTTCTTTCCTCAAAATTGTAAAAAAAAGAACTCTTTTCC<br>AAACAAGTTGG**ATATAA**ATAATTGAATAGACAAATAAATACTGCGGGCGCACCATATTTT<br>TGTAAGGAAAGGGAAAGAATCATTATCTCATGTGGTGCTGGCGGGCAATGCTAGCCCTAC<br>GTTACAACATAACATTCCACACTTTTTTCAACAAGATCGAGAAAATCCAATAAACAAGTAGA<br>AAAAAACCCGCCGCTCAACGCATAAATAGAGC**TATA**GCCGGTTCTCTTCAGCATAGTCAGC<br>CAATTCCCTTCAGCACAGTCGGTAACAATATCACGAGGTTTGCTACGACGGTGCGGCCCAT<br>T**TATA**CCATACGACTCTCTTCTCT**TATA**AGGGAGATAAGATACATACCCCCTTTTCCACCAA<br>AGAAAATGAGCCGCGATCTCAATTTCTCTGGATTTAACCAATTGGGTCGCCTCCAACCCATG<br>CAAGGGCTTCCTAGGGCGTCGTCGTCGTCAACCTCGGCGTCGAGAGATCGAGACGGCGGC<br>GATGGCGATGGCGGCGGCGGCGGCGTGACGATGACGAACGGGC
106	41277400	41277993	594	-	CTTGCATTCCAAACATGCCTAAACTTTTCTTTTGTTA**ATATATA**CTACTCAGAATGCCATAT<br>GTGCATGTCTAATGGTCCTTGATAATTGGTCCCAGAGCCACAATCATGTGGTGAATATCATA<br>ATTAAATCATACAGAGAAATCATGTGATGCACAACTAAACCCAATTAATAAGGAGAAACCA<br>TGTGATGCACAACTAAACCCCTAATAAGGAGTAAATATCCCTGATGAACATCACTATGTTAG<br>CTGTACTCACTAGTTCTTGACAATTGGTCCAGTGCCACATCACCCCTCTTATCATCATGGGTT<br>GCCATGAGACAAAGGCATGATGGCATTTGGGACTAAAAACCTCACCCTGTTAGTGGGTCTG<br>TGCAGGGTGGGCAAGTGCAAGAGCTAGGTAGGGGCAGAGATGAGAGAAGCTAGCTAGG<br>GAAGGTGGTGGAAATTATGCAAGAAATTTAAAGAATAATTGGC**TATATAT**GCAGGGGGT<br>GTAGGCTAGGCCCATCTCTACTCCTGTCTCTCCTTCTCACTACATTCCACCCTCAATCCTGCC<br>CCCTTGTCTTGCTCCCAGCCCCTGTCAGGGAGGAACAGCAGGT

107	41313576	41314170	595	+	CGATCGAGAGAGAAAGCATCGCGTT**TATATA**GAGCTCAAAACCAAAAGGCAAATCTGTGC<br>TCG**TATA**GCTATTCAAACAGCCACCCAAAAGAAAAGCGTGAAGTGCGCATGACTCCACGC<br>CATCCGGAAGGCAAGAAATCAGGAAGAGACCACATTTGCGATGGCGGCGAGATGCAAGC<br>GTATGATCCAACAACCAAATATTGTTGGTTAAAGAAAAAAACAAAAAAAAATAGTAATACG<br>TTTCGTCTGTAAAATTAATTAAGACTCGGCAAAAGAAACGAAAAGATGCAGGCGATAGATA<br>AAAGTCATCGAGGAATCGCGAGGCGACGAACTAGACCAAGCCAGCCGCAAGGAACCGCG<br>GTGGTGGGGGGGAGGCCGTACCCGCTGGGGAGCGCGTCGACGAAGGTGAGCAGCATCTT<br>GAGCTTGCTGCCGCCATCGGACGCGAGGCCGGCGTGCATCTCGACGACCATGGCGTCCAC<br>GACCTGGCGCAGCCTCGCGGGCGGCGTGGCACACCCCTCCTCGAACTCCCGCAGCAGCGC<br>CACCGCCCGCCGCCACCGCGCCCGCGCCGACGCCCTGCGCGCCACGAGCGCCGCGG
108	41328214	41328798	585	-	AATTCGTGTGATTTCTTCCTAAAATCATAGGTGAGATCTACATAGGGAAGTTCAAT**ATTAT**<br>**A**TCTCTTCCTC**ATATAT**TTCTCTTGGTTGGTCAGTCTTTTTTGTTACACTAATTTTTCTTAGC<br>GCTTTCTTTCACGTGGG**ATTATA**AGGAAAAAAAAGGATAACGACTGTGACTACTTTTAAGA<br>CAGTAGAGATACGTACTTAAATTTAGAAGATGTTCCGATCTCCTGTGTAATTACTGCGGGTT<br>CTTTTACTTGAAAGAATGCCTTGATTGAGTCAGGACTTCTGCGTGCATTGGTAAGTTTGAAG<br>ATTGTGTGGGCATGGGTGATGGATGTGCCTGTCCATGATACGGTTGTAAAGCCGACAAAG<br>CCTCTCATTGGCCAGAGGACAAAGAGCTGGCCTGTACTTGCAGCCTGGCCTAAACCCCCCA<br>GTGATAAAAAAAATTCACAACCATCAGTAAATTAAAAAAAAAAAACGCTCCTCACTGATCAG<br>TCAGCCAGTCCATCCATTTTCACCTTGTCCAAAGTCCAACTGCAATCCAGCTCACCTGCAGTC<br>TGGACCTGTCCACTGTCCAGCCCCTCCTCGCT
109	41761766	41762351	586	-	CTTCCACCGTTCTTTTTAACTTGACACTACAAGTAATTGT**TATA**AAAACTTTGACAACGTACT<br>CATCTTTTATTAAAA**ATTATA**AATACTAAAAAGAGGTTGTATTATCAAAGTGGAGTACTTA<br>ATT**TATA**ATCATCAAAACATAATTCTACTATTTCCATTCATCAAAACATTAATTAAGAGTTTT<br>GCTTGTGGAAAGATAAAATCTCACTAATCAACAATGTCAAG**TATA**TGGGAATAGAGGAAT<br>ATTTAACTAGGCAAATTAAGTAATCATTTTTTTCCTATTTGACCAAACTAATTAGTACATTGG<br>AGGGATTCCAGTCACAGGCTGTCTGCAAAAATCGATTTTCACAAATGATCACT**TATA**AGAT<br>AGGGAAAATTGTAACCAGCACTGCTTGTGAAAAATAATGTCCATACTAAAAGATTATGCGA<br>AGTCTCATACCCCTCTCGGCCACGCTAAGTCCTTTACTCCTCTCTTCCTGGCTCCCCAACCCT<br>ACACCTCACCTGCTGCCCAACTCTTCTCCTCCCTCTTCTCTCTCCAGGCCATTAGCGCTTCGG<br>CAAGCGCCTGCATCCTCCATCCCTAGCC
110	41767406	41767983	578	+	AATATTTCCAACGGCAAATGGGGGATCGGGTAGTTGGTAAACATAGGAATTGTTCTTACAC<br>GGACGGAACTGGGCCAGGCAGAAGTCTAATCAGGCGCCGCCCTATCTCCGGTAGGTCGTC<br>CTTTAGCTTTGCACCACGGGAGCCGTGGAAAAGTCTGACGAGGCTGAGCGTTTTCGATCGA<br>GCCTCGTGCATGGTCCGCGGATTAATTTGTAGCGGAGCAAAAAAGTTGCTTGTTCCCGGGC<br>AATGTCAACCGTCAACGATCGGGTTTGTAGCGCACGCACGTAAACACTGATGGTGTTTGTA<br>CGCAAGTGGAGTTACGGGGAATCGCGCCGTTAGCCAAACGACCGGAGGCGCATGCATGC<br>AGGGATGCAGCCCGCAGGCAGATAGGCCATGCATGCATGAAACTCGAGCACGCAAACGCA<br>AACACGCAATCCACGCCTCGCCGCTGGCCCCCGCGCACGCAGCCCTGCCCTCCGCGGAGCC<br>GACCTGTTGCCTAAACGCAATTTAAAGCCCCCCGCGTGGGCGGGGCCGCCTCCCTCCCCTC<br>CGCGCTCGCTTTCGTCGCCTTCGCCCGCGCCGA
111	41811964	41812551	588	-	AAAAATGCTTATTAAAATGAAAAATCCGGTAGTACATGTCTACATAGCAATGGGCAAAATA<br>CATAATTAAGCTGAGTTCTTGATGAGACGATAATAACCTGTAAAAAATGCTAATTAGATCG<br>GGAAAAAAATTGATGACTTACTTGACATGATTTCAGTACTAGTTAAACTCCTGATCCTCAAG<br>TCCCGCCAAATTCTTTAGACCTTCTTTTCAATAAGCAAAAC**TATA**ACCCCAATTGCAAACCC<br>TAACA**ATATAA**TTCAATTCAA**TATA**GGGGAATCGGTAATCAATCAATTGAAACACTATCTT<br>ATTGCTTCAAAAGAAATTAATTTGCACTAAAATTGGTCAAGAAATGTTTTCATACCATCACA<br>ATGCAGAAAAAGGGAGGAGACGTGGAGGGCTTGATGCCTTGATGCCGCAACCTGCAATCA<br>GCGATTCCTGCTCGATGAGGCGTGCTACCATGCCGTGCTGAGCACCTGAGCGGTGAGCCG<br>GTGGCCAGCGATCATGGCAAGTGGGTGCGCCGTGCAGTGCCTCCGCTCCGCCGCTTCCAG<br>CTTCCGCCGTTCTGCGATTTGGGGGCGCCGGGCTAGGCG
112	42043135	42043730	596	+	TGCCAACATGAAATTGGTAGTGTAGAAAACATAAGTAGGATACGTGAAGGTTTGTATGCG<br>TACTACTCCATCCTTAAAAAAAAACCTGCCTTGTACTGAGATTTAACATCCTAACACAGTGA<br>ATTAGAGTTTAGACGTAGCGTATGTTTAGATTCATT**ATTATA**TCCTAATACGATGTTTTTTT<br>TTAAGATGGGGATGTATGTATGTGTGGAGAGAGGGGAATAGATACTTCCGGTTGTTGTCA<br>TGGCAATCATTGTAGAGATCTATGGACGTCAATGAATAATTGAAGTCCCTCACGGCTGGAT<br>GACCAAAGGTGCGTTCAAGCATGAAACATGCCTGCATCCATGCCAGT**TATA**AGTAGACGT<br>GCAGTACAGCAACGTCCAAATCCAAGCAATCTTTCGATATCTAGTCATAGATTCAGCTTCGT<br>ACACGGCATCTCTAGCGGTCCCCCAGTGGAAGGCTAATCCGACACCTTAAACACAGAAACG

					TTCACATGAACAGCAGAGAAGAAACACGAGTCCAAAAGTCCAAACCGGCCAAGCCACCAC<br>TGGAGCGAAGCTGTAGCGTGCGCCGCAAATGGGAAAATTAGGCCGCAA
113	42055217	42055716	500	-	TTTTTCTGCGTGAGAAAGACACACCGGGGATGGATGATCACGTGAAGTTGGGTCGAGCAC<br>CGCGATGCAGGCAGGAGCAGGAGTGGAGAGGAGAGCGTGTTTTTGCAAGTGGAGTAGG<br>GGACGGCGCGGGGACGTGTCGGCGTTGAGGGAGGCGACGGATAAGACGGCTGCGGTTG<br>GACCGGAGGCCGGCTGGTGCAGTGCAGTGCAGGCTTTAATAAGGTAAGGCAGGGGCACG<br>GCGGGGTGGTGTTGGACCTGCGCGTCCGGTGGCTTTTGGCAGGGTTTAGTTATTGTGGTC<br>GTTGTCAGGACGCGTAGATAAAACAAAAGAAAAGAAAGGCGGCCAGCGCCGGCTGCGGA<br>TGCGCCTGGCCCGGTCCCCTCCAGCTCCTGCCCGCGCGGGGCTCGAGGCGCCCATCCGCAC<br>AGTCTCTCTCTCGCCACGCCGCGCGCTCTCCTCGGGAACCGCCGCGCCAAAGCCCTCCTCTC<br>CTCGTCGTCGCCGCGCCGCTCGA
114	42146934	42147523	590	-	GTTCGACCGAGCTGAAATGTGTGAGAATGTAGGGAACCTTAAGCATAAAAAAAAAGAGAG<br>TAGAGAACCTCGTACCTGCGACTTTATTCAATTTTTTTCACGGTCTAGCGTTTGGAGCCGGA<br>AATTATTCTAGCAGTTGCATTAGCAAAAACACTTATCAAAATATTACTAACAGGCAGCGAA<br>GCAACACAAATACATCTTAGCTAGCAAAGTAGTTGCATTAGCAAATTTATTTACCAAAATAC<br>CGAACTTATGTTTACCAAACAAACAACATATGTTTACCAAACTTTAAATAACACCAAATCTCC<br>TGTTTAAAATTTGCATTAGCAAACCATCCGAACAGATCCGAAATGCACGTTGACGAATGCG<br>CGAAAGCATTAATTGTTCAGAAACAGCCCCTGAAGGCTGAAGCTTAGTACGGTGTGTTGCG<br>TCCAGTTGGAACTTGGAACCACCTCTTTCCAATCTTGTCCCCGTTCGTCCGGTTATCCGGTGT<br>AGTGCGCCAGAAGAAGGGAGGAAGGGAGAGGGAGAATTCGGGGGACCACATTCCAAGTT<br>CCAACGGAGGCCGGAGAGGATGGTCCATGTCTGATGTC
115	42310412	42310990	579	+	AGGTGGAAAATTGGCAATTAAACATGGAATTAAATTTCAATAAAGGCCTGGTAAATAGGTT<br>TGGGATTTAAACAGATTCCAATAGCAAAAGCATCAATCCAATTTCCTGTACTAATTCCTCGT<br>TAACACAAATCGTACGGACTT**TATA**AGCTTTTAAATTTTGCTAACGCAAAAAAGCTGATCA<br>GAG**TATA**ATATTCAAAATCTACTGCATCGACAATGAACAAACCACGAGTATGTTCAAAAAT<br>GGGGAAAATGAAGCGAAAAATCTAACACAACAAAAGAA**ATATAT**TAGATGAAAAATATG<br>CTCAACTTGTCAGACTTCTCGGTGCGGTACGTGCGCTCGCCCAGCGAGAGGCACGCGATGA<br>ACTTGTCCTTGAACTGCATATCCGCCTGTGGCCACCAACCAAATCAAAACAACGCG**TACAA**<br>**AACCA**CAGCAGCAGCGTACACAGACGGAATGAATCAAAACCACCGATCCCCAATTCCACCC<br>CCCTCCGCTTCGCAGCCGCAAGCACACACACCCAACCCGGCCCCGTACCTTCACTATCCGGA<br>TGCGCGCGATGCCGGCGAACTCGTCGACGG
116	42325296	42325885	590	+	GCCTCCAAATAGTATCACTTTGATTAATTACTAACCCTACTAGTATCTCTATGCAGCTTGGAA<br>CATGCACAATCAATTTCG**TATA**TTTTCAAATCCATATTTCCCTAATCAAACTATTTCATCAA<br>ATTTTGTCGGTGCAATCTGGGCAATTTCAGTACTTTGCGAGCATT**TATA**CT**TATA**CAAGAA<br>ATACTCCGTATGAAAGAAATCTCAGTGGTCAACCCTAAATTAAATGAAACACAAGGGGGTG<br>AAAGCGAAAGGAGATGAACATAAAACATGAAAACCAAGGGAGAGAAAAAAAAAAAGGTT<br>CAACTACTCTTTCCATTCTCGCTCGCGGCGCCTCCTCCTCCTCTCCCAATTCCGCCGCCAATTT<br>CCGGTGCCGACGAGGAGTCGGAGTCGGAGTCCGCCTCGCCGGCGGCGTCCGAGTGTGTGT<br>GTGTATGTAATGGACGCCGACGAGCCGCCGGAGCACAGCACCGACCCGCTCACGCGCCGC<br>GCCTGCAGCTGCTGCTGCAGGTAGGGTGTTCGACGCATGGGCTGCACCACCTCGCACGAC<br>GCGTTCGCCGCAGCGAGGGCGCGGGCGCGGGCGTCGTCG
117	42579010	42579591	582	+	ATTAAGGATTTG**ATATAT**CACAATTGACAGTAATTAATTTGCTTGCTGTGTGTTTTTATGAA<br>CTTCCTTAGCTGATCATGTTTTCATTCCATCTTCACCTCGTTTTACAGCTGCGAACGAATTCA<br>GAAAGCATTAATTAAGATTCAGAGGCTGCAGAATGCAAATCAAGCAACAATAATGCATACT<br>AATATGGATTGCAAACACTCTGGATTACAACTAAGTTTTGATCTACGTTTTTACTCGTTTCCT<br>GTTTAGTTTCTGGTAATTTCAGGAAGCAAACACACCCATCTCGTTTTGATTCTTTCCTCTGGA<br>TTACGACTAAGTTGGCAGCTATGTCGTCGTCGGCGGCGCCGGCGAGGAAAGAGAATGGCA<br>CCGTCGCCGCCCCCGCCGCCTCATCCGCCGCCGCCAGCAACACGTTCCACGTGCTCAACTCC<br>GGCAGCGCCGCCGCCGCCGTCGCAGCCGGACGTGCGTATGTTCAGTCGCCGCGAGCGACG<br>GCGCTGCGGTCGCCGCAGGTGCGAGCCATCACCAACTACGGCATGTTCCCGACCACCGGC<br>GCTTCGGCGGCGAGGACGGCCATGGCGG
118	42580724	42581312	589	-	CCAAGATTTGATCATGAACTGGACATGAACCAAAAAATGTAAAATAGTAACCAAGGTTTGA<br>CCATGAACTGAAAAATGTAAAATAGTAACCAGGATTTGACCATGAACTGAAAATTGTAAAA<br>CAGTAACCAAGATTTAATCATGAACTGGACATGAACCGAAAAAAGTAAAACAGTAACCAA<br>GATAATCTTGACCATGAACTGAAAAATGTAAAACAGTAACCAGGATTTGATCATGAACTGA<br>ACATGC**TACAAAA**TCAATCACAGTTTCTGTCTGAAAATTATTCAGTCTTGTGACCTGGATG<br>GCGAGGCAATCTGGATGGAAGGTGGAGGGGCAGGTGTCGCAGCAGAGCAGCTGGCCGCC<br>GTCGGCGCAGACGCCGCAGGCGTCGTCGCTGCAATCCTTGCCGCCGCCGTTCTTCTTCACCT<br>GATGACGAGGAGAAGAGGTGGAGACGACGACGACGCCGTCGAGGAGCTGCATCTTCTTC

					GTCTTCGCGAGCTTCCTCTTGGCCTGCGAGCTCTTCTCCTTCTCCTGCTCCAGCGACGCCATG<br>GCCTTCTCCCGGGCGCGCATGGCGGTCACCCGCTCCTGCGCC
119	42606697	42607289	593	-	GCATTACCCTAGCAAATAAAATCTGTTAAAAGAATGG**TACATAAA**AAATCAATAACAACA<br>GAACAGGCAATATGGAATAATAACTACCTTTGTGTTGGCAATAAAAGCCCTTTGAACAGCA<br>AGTGCATCAGATTTACGATCAGTTTCTGATGAGTTCAATGCATCAAACTCTGGTTCAGCAGA<br>GATAAAGGAGCCATTTCTACAAGAGAAATAACGAACTTTTTAGAACTGCATTTCAT**ATTAT**<br>**ATATAA**ATAAATAAGGAGATAAAAATCATCATGAGGATAACCTATTTTCCTGAACTGTCCA<br>CCCTCCTTCTCCATCCAAAGACAAAACGATGTCATGAAATGCAACTAATGCTGGACGATGA<br>GAGATTGTTATGCATGATGTGCCCATTGCTTGAACCCTTTTGCAGAAACGCTCTTCCATATC<br>AGTTGTCACAGCACTAGTGCATTCATCCAGGATGGCAAACTTTGGCCTATGGTAGAACAGC<br>CGGGCCATTCCCAATCTTTGTTGCTCTCCAAGAGACAATTCATCACCCCAGTTAACTTCCTTA<br>TCAAGAGGGTAGCGTTCTAGCAAATATTCCAGATCCACCTA
120	42661179	42661758	580	-	TGCAACAAGTTTTGGAAAAGGT**TATATTATATA**GATATTGGAGCATAAGTACCTGCTTGA<br>TCAACCGAATGTCTCGTAAAGCTTACACCATATCTTAGGATCCCATGTTTCCATTTTTTGGAT<br>GAGCCTTTGCCATGTTTTGCATTCCACCTATTAAAGTTAAGGTGTAAGACACATAAAAGGAT<br>CAGAATAAAAAA**ATTATA**GAAAAGAATATTTTCAATTTTCACTACCTTAACACTTTCTGGCA<br>ATAGCCACCCAATGCTTG**ATTATA**TTTTTGCACAGCTTTAGGTAGTGATATGGTTTCTTATCA<br>TCGTAACCAGATGGGTGGCTTCTTGTTTGGTCATAACTTATCATCTCCTGAAGAGGGGATG<br>GTAAATTCCCCAAGTAGGTCACAGGTGGCCACTGTATGAGGTTGTTAACCTCCTGCTCTACC<br>TTATGTGGGTTCATACACAAGACCTTCAACAAGTATTTCCCCTTCTCAGTTTCATCCCAATCC<br>AACGGATACCTCTGTAGCGTCTTCACATTGCCCTCTGCAATGTAATCAAGGAATCTCACGAG<br>GGGCATGTCTTTGGGGGAATCCA
121	42668181	42668772	592	-	CAGGTTGAATCTTTATGATTTTGTATGTTTCACTGATTAACCTTTGACATTATTTAATTGAAA<br>TTTTACCATAGTGGAA**ATATAA**TTTTTTGGGGCATATTTACCTTCTTTGTGTTAACTTTCTTG<br>ATGTAAGGCCTGTCATGTTGTAGCAAAAAGAACATTTCAA**TATA**GAAAACATCTGGATTTC<br>TTTTTGCATTGCTCATTTTACTTTCAGGACTTTATTTGAAAAAACATAAATGCTTGTATTATTG<br>TTTCAACCACAGTGTGTGGAAAAATACATGTTTGCTTACAGCAGTAACCTTATTGACACTGA<br>ATTGGAGATGTTACATGTTAACCTTTGAAATCTGAACCTGGGATGTGTGTGTGTGGTGGTG<br>GTGGTCAGGCCAAGAAGAAGATGGCGAGGCTGATACAAAGGATCATCCAGGACAAGAGG<br>GCAAGGAGGGCCGCCGCGGGACGCCATCGATGTCCTCATCGGAGACGGCAGCAATGAGC<br>TCACCGACAAACTCATCTCTGACACCATGATCGACCTCATGATACCCGCAAGGACTCCGTCC<br>CGGTTCTCATCATGCTCACCATCAAGTTCCTCAGCGAG
122	42734129	42734632	504	-	GAAGTTTTGAAGAAAATTTTGAAAAAGCAAATAATCTGAATTGCAAATCATTTAAAAAATA<br>AAAAATAAAAACTGTACAATATCACGAGCATTGACTTGAATATGAAGTTTTGAAGAAAAAT<br>TTGAAAAAGCAAATAATCTGAATTGCAAATCATTTTGAAAAAAGAAGTAATAAAAATTATG<br>TTATGAAGAAAAATTTGAAAAGGCAAATAATCTGAATTGCAAATCATTTAAAAAAAATCAA<br>ATAATCTGAATTGCAAATCATTTTAAAAATCAAAAAGTAAAAAGAAATCAATTAATCTGAAT<br>TGCAAATCATTAAAAAAAATCAAAAATCATTTAAAAAATCAAAAATAGTGCTGGCTGTGGG<br>GTTCGAACCCACGCGCACTTCTGTGCAGAAGATCTTAAGTCTTCCCCCTTAACCACTCGGGC<br>AAACCAGCTAAACGACAGTGATGGTCATAAAATGTTCTAGTCCTTGAAGCAACCGACTTTG<br>CTGCATGGATGTCA
123	43058624	43059216	593	-	AGTAGGAGTAAAAAAAAAAAAATACTCCGATGACTTGAACAGGGACGGACCTATGATCGGC<br>CTAAGGACCGAGCCAGCCCATGAAGACTTAGACCTTGTATGCTGTTTACATTAGGCCTATT<br>GGGCCGAACGAGACGACGTATGATCATGTTTTGGGCCTCCTTTTTGGAGAAGGGAAGGCC<br>CACA**TATA**GATGTGGTGGTCGTCTTGTTGGTGGTGACAGGTCCTAACCTAGTAACCCTACA<br>CTGTCACAAAATTCACAATCGATCGAGGGATCCCAGGCGACGGAGCTCCGGCGAGCTCCG<br>GCGGGCGTCGGCGTCGGCGTCGGCGGCAGTTAGGGCGAGGCTATCTAGGGCATCAGAGA<br>TCCGGGATGGCGGTCCAGGTCCAGGAGTCGAAGCGGGTGGGGAGGCAGCTGCCCCCTCA<br>CCGGCCACCGCGATCCGTCTCCCTCGCCGCTTCCATTTCCTTCTCCTCCGCCCGCAAGCCTCC<br>CGAGCCGCTCCGCCGCGCCGTCGCCGACTGCCTCTCTCCTCCCGCTCCCCACACCCACACCC<br>ATGCCCCGCCACCCGCCGCTTCCTCTGCCCCCGCCGAGGCCTCCAGAA
124	43101914	43102493	508	-	ATCCCCTGGCCCTGCAATGAAGTAGTAACAACCTCCATCTTAAAAATCGTAAGCAGTGGCA<br>AGCACTATTAAAAAGGATTTTCATGTGTAGGTCCCTTTTACTTTTACTAAGGAGGAAAAAAA<br>ATACCCGCT**TATA**AAAATGTGCGAGAGGTGTTTGATCAAGGCCCACATTTTAAATTTTCAC<br>AAGTAGGTCTCTTAACAGATCCGTCTACAATAATAAATTATTTTCCCAGACGAGCATCTTAA<br>GAGGTTTGTATGTGAAAATTGAGGCACGACTGGCCGGCTCCATTCCCCATTATTTATTTTCT<br>TTCTTTTCTCCTTATCAATCCATTTCACTTCTTCTCTCTCCCCCTTCACTTCTCCTTCTCTTCTCC<br>TCTCTTCCCCATCGAGCAATGACAGGAGCATTGGTGCCAGGAGCGGCTAACTATGGGAGC<br>GATCGC**TATA**GAGGTTGGCGGCGACTGTGGAGGCCGACAGCGGTGACGACGACAACAGA

					GATAGGTGGTGGCACATCTCCCTCTTCGATGACGGGAGGGGAAGAACCATCGAGGAGGG<br>CCCTAGCCATGGTGGATCTGTCTCCTGC
125	43107547	43108108	562	+	ATTTATGTTGCTCCTCAGTTGTCTTTTTCTCTGTGCAACAGCTCCGATTCCAGGCTCCCTGTTT<br>AGACAGTATGATGTACAAGGTCCAATTCCAGT**TATA**AAAGATGTTTTTATCCCTCCAGACA<br>CAGAAGAAGATGGGCTGCCTGCTGCCCTTGAAGATGCCATGCCTCTAATTTCTTTTTTGGCC<br>CCTCTATGTAAGAAGTGTTACAGTTTGTTTAGTCAATTTGAACATGTAATAATGACAAGATA<br>AATTTACGAGCAGATCGACACATGCAAAGATCGAATTGCTGCTGCCCTTGATAAATTGCGA<br>TTTACGACATACATCTTCGTGGGAGCGAGGTGCTGCTTCTGGGCAGGAACCTGATACTGTT<br>TTACTTGGTGGCCGAGGTTTTGGAGCAGCCATGTCTGCTCTAACTGACGCCCTCCCCCGAC<br>GTCCTGCTTCTCTCCGCCGGCCGCCTCCTGCTCTGCTCCCGCCGTCGCCTCACCCGTGTGAA<br>GCCGCTACACGCCGCTGGCGTCGCTGCCGCTGGAGCCGGGTCCACGTCCCGCCGCCGTCG<br>AGGTCGCC
126	43217504	43218096	593	-	CACCCTGTTAGCATATTATGGAGGAAAACAACCAAACCAAACGGCACTCTTGAACCAAGAA<br>G**ATATAA**TCACAACAAATTGTGAATACAAATTTAAAACAACAAAATTGATTTGATACCTAT<br>GCATTGAATATGTGGGAGATTAAAGAAGTAGTGACTTTAATAC**TATATATA**GCATCCAAA<br>TGTTTACAGCATCATCAAGTTAAAGGATCGATCAGAGGGGGAAGGCGTGTGGGTTAGAAG<br>AGGAGACAAGGAAGGAGGAGGATGTGGGTGTGGTGGAAGCTGACGCGAGTACAGGTCT<br>AGTCGAGTGGAAGCCATTGTTTAAGCTGATCATGGTGGTCATGTTTATGGAAGCAGATGA<br>GGTGCCAGTGTAGGAGGTGGAGGTGGAGGTAGAGATGGAGGCGGAGGAGGATTGGCGC<br>GGTCGGCGGCGTGGTGCAGGGCCCTTCTCGGCCCAGACGAGCTCCTGCAGCTTGACAGCC<br>TGCGCGTCGGGGTAGTAGCCCCAGAAGGCGCAGGTGCAACAGGCGGCGGAGGGCGTGA<br>GGTTGGTGCCGGCCCGGTGGATGTAGCGGTGGCTGTACCTTCTTCCCAAGCAATCGA
